# Effect of a glucose impulse on the CcpA regulon in *Staphylococcus aureus*

**DOI:** 10.1186/1471-2180-9-95

**Published:** 2009-05-18

**Authors:** Kati Seidl, Susanne Müller, Patrice François, Carsten Kriebitzsch, Jacques Schrenzel, Susanne Engelmann, Markus Bischoff, Brigitte Berger-Bächi

**Affiliations:** 1 Institute of Medical Microbiology, University of Zürich, Zürich, Switzerland; 2 Service of Infectious Diseases/Genomic Research Laboratory, University of Geneva Hospitals, Geneva, Switzerland; 3 Institute of Microbiology, University of Greifswald, Greifswald, Germany; 4 Institute of Medical Microbiology and Hygiene, University of Saarland Hospital, Homburg/Saar, Germany

## Abstract

**Background:**

The catabolite control protein A (CcpA) is a member of the LacI/GalR family of transcriptional regulators controlling carbon-metabolism pathways in low-GC Gram-positive bacteria. It functions as a catabolite repressor or activator, allowing the bacteria to utilize the preferred carbon source over secondary carbon sources. This study is the first CcpA-dependent transcriptome and proteome analysis in *Staphylococcus aureus*, focussing on short-time effects of glucose under stable pH conditions.

**Results:**

The addition of glucose to exponentially growing *S. aureus *increased the expression of genes and enzymes of the glycolytic pathway, while genes and proteins of the tricarboxylic acid (TCA) cycle, required for the complete oxidation of glucose, were repressed via CcpA. Phosphotransacetylase and acetate kinase, converting acetyl-CoA to acetate with a concomitant substrate-level phosphorylation, were neither regulated by glucose nor by CcpA. CcpA directly repressed genes involved in utilization of amino acids as secondary carbon sources. Interestingly, the expression of a larger number of genes was found to be affected by *ccpA *inactivation in the absence of glucose than after glucose addition, suggesting that glucose-independent effects due to CcpA may have a particular impact in *S. aureus*. In the presence of glucose, CcpA was found to regulate the expression of genes involved in metabolism, but also that of genes coding for virulence determinants.

**Conclusion:**

This study describes the CcpA regulon of exponentially growing *S. aureus *cells. As in other bacteria, CcpA of *S. aureus *seems to control a large regulon that comprises metabolic genes as well as virulence determinants that are affected in their expression by CcpA in a glucose-dependent as well as -independent manner.

## Background

*Staphylococcus aureus *is one of the leading causes for nosocomial infections. It has been the subject of intensive research for many years and there is a large amount of data available concerning the regulation, function, and structure of various virulence factors. Recent studies suggest that basic physiology determines not only growth and survival but also pathogenicity and adaptation to environmental conditions. Therefore, more knowledge about cell physiology and molecular processes involved in infection is necessary to better understand staphylococcal pathogenicity.

One of the important and highly conserved regulators of carbon catabolite regulation in low-GC Gram-positive bacteria is the catabolite control protein A, CcpA, which has been intensively studied in *Bacillus subtilis *[1,2]. In the presence of glucose or other rapidly metabolized carbon sources, CcpA is activated by complex formation with the corepressor Hpr that has been phosphorylated on residue Ser46. Hpr has dual functions; it can be phosphorylated either at Ser46 or at His15. In the latter form, it acts in the sugar phosphotransferase system (PTS) for sugar uptake. The CcpA(Hpr-Ser46-P) complex has an increased affinity for particular *cis*-acting sequences, termed *cre*-sites (catabolite responsive elements), and thereby represses or enhances gene expression, depending on the position of the *cre *in relation to the operator sequence [3,4]. These *cis*-acting DNA sequences have been extensively studied through mutagenesis [3-8], however, the consensus sequences differ slightly from study to study. In *B. subtilis*, a second corepressor, Crh, which is highly homologous to Hpr, but can only be phosphorylated at Ser46, can also form a complex and thus activate CcpA [9]. While *S. aureus *possesses a HPr-homologue, no Crh-homologue can be found in this organism [10].

CcpA has been shown to play a similar role in controlling metabolism in other bacteria, such as *Bacillus cereus *[11], *Staphylococcus xylosus *[12], *Lactococcus lactis *[13], *Streptococcus pneumoniae *[14], *Streptococcus mutans *[15], and *Listeria monocytogenes *[16]. In addition to its role in metabolism, CcpA was reported to regulate the expression of several virulence factors and to be involved in antibiotic resistance [14,15,17-24].

The aim of this study was to gain a genome wide overview of the genes and proteins subject to CcpA-control in *S. aureus *during exponential growth in a pH-controlled environment, in the absence of additional glucose and 30 min after glucose addition.

## Results and discussion

### Physiological characteristics of the Newman wild-type and its Δ*ccpA *mutant

**Figure 1:**
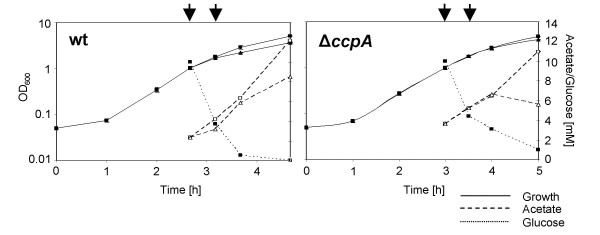
**Growth, glucose consumption and acetate build-up**. Growth, glucose consumption and acetate formation in strain Newman (wt) and its isogenic Δ*ccpA *mutant (Δ*ccpA*). Cells were grown to an OD_600 _of 1, cultures were split and 10 mM glucose was added to one half of the culture (squares), while the other half remained without glucose (triangles). Cells were sampled at an OD_600 _of 1 and 30 min after glucose addition for RNA isolation (indicated by arrows). Experiments shown are representative for three independent experiments.

The transcriptomes of strain Newman and its isogenic Δ*ccpA *mutant MST14 were analyzed in LB, a complex medium essentially free of glucose and other rapidly catabolizable sugars [25], under controlled pH conditions in exponential growth (OD_600 _of 1), and 30 min after the addition of 10 mM glucose. In the absence of glucose, the wild-type had a slightly lower doubling time than the mutant (25.7 ± 1.29 min versus 31.8 ± 1.29 min, *P *< 0.01). The addition of 10 mM glucose at OD_600 _of 1 increased the growth rate of the wild-type but had only a minor effect on that of the mutant (Fig. 1). 60 min after glucose addition, glucose was depleted from the medium down to 0.3 mM by the wild-type, while still 3 mM of glucose were left in the culture of the mutant (Fig. 1). Despite increased growth and glucose consumption rates in the wild-type culture, acetate production was only slightly enhanced compared to the mutant, in line with previous findings [24]. No lactate was excreted under these conditions at any time point sampled, confirming the aerobic growth conditions. Acidification of the medium upon glucose metabolism was prevented by HEPES-buffering, which allowed maintaining the pH of the growth media at 7.5 for both strains and under both growth conditions for at least 2 h past glucose addition.

### Transcriptome analysis

The total number of genes, which were expressed at a sufficient level to give meaningful data, was 2479. 111 of these genes had no homologues in strain Newman, and were therefore excluded from the analysis. Of the 2368 remaining genes, a total of 155 were found to be affected upon glucose addition in a CcpA-dependent manner, while 21 genes seemed to be controlled by CcpA and other regulatory proteins at the same time in the presence of glucose, and 10 genes exhibited CcpA-independent glucose effects. The largest group, comprising 226 genes, however, was affected by *ccpA *inactivation even without glucose addition to the LB medium (Table 1). While regulatory classes partly overlapped, the overall range of differential gene expression was only narrow, peaking around 2- to 3-fold induction or repression.

**Table 1:** Numbers of *S. aureus *genes subject to regulation by glucose and/or CcpA^a^

Regulatory class^b^	Number of genes	Genes associated with putative *cre*-sites
*CcpA-dependent in the absence of glucose*	**226**	**38**
Lower expression in wild-type	118	28
Higher expression in wild-type	108	10
		
*CcpA- and glucose-dependent*	**155**	**48**
Down-regulated	81	38
Up-regulated	74	10
		
*Partially dependent on CcpA*	**21**	**3**
		
*CcpA-independent, but glucose-dependent*	**10**	**0**
Down-regulated	10	0
Up-regulated	0	0

^a^A gene was considered to be regulated if transcription was induced or repressed at least two fold

^b^Classes partly overlap

**Figure 2:**
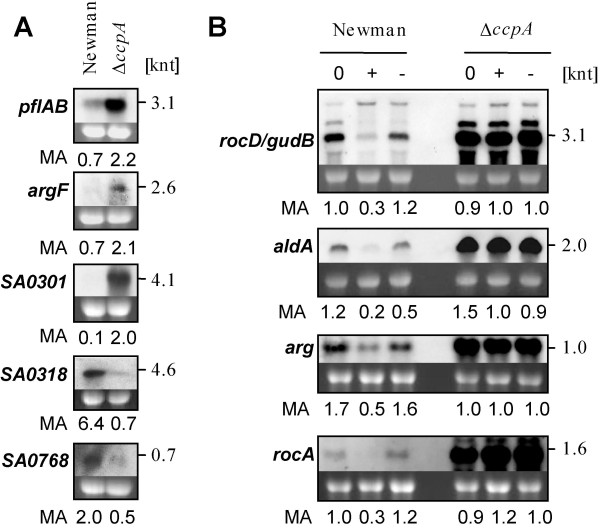
**Northern blot analyses of CcpA-dependent genes**. A, Transcription of genes showing differential expression in the *ccpA *mutant in the absence of glucose. Gene expression at an OD_600 _of 1 in strain Newman and its Δ*ccpA *mutant is shown. B, Transcription of CcpA-dependent, glucose-dependent genes in strain Newman and its Δ*ccpA *mutant. Cells were grown to an OD_600 _of 1, cultures where split and glucose added to one half (+), while the other half remained without glucose (-). RNA was sampled at an OD_600 _of 1, and after 30 min. RNA loading is represented by the intensity of the 16S rRNA. Data are representative for at least two independent experiments. MA, microarray data.

In order to support our microarray findings, we analyzed the expression of five genes showing differential expression in the Δ*ccpA *mutant compared to the parent strain in LB in the absence of glucose, and of four CcpA- and glucose-dependent genes by Northern blot analyses (Fig. 2). The tested genes showed the same trend in expression by Northern as in the microarray.

#### CcpA-dependent differential gene expression without glucose addition

**Figure 3:**
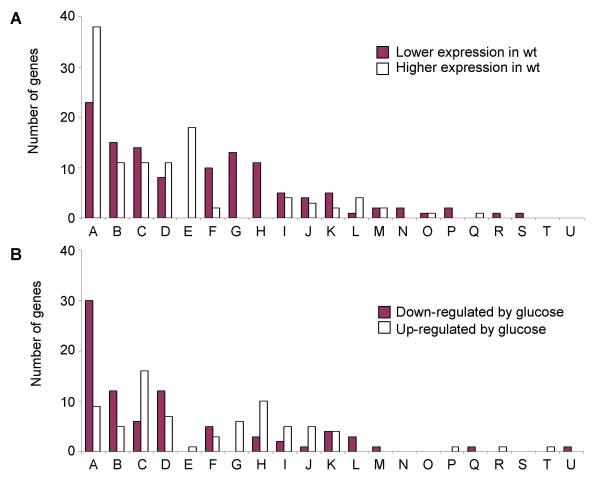
**Functional classes of CcpA-dependent genes**. Functional classification according to the DOGAN website [26] of genes that were found to be regulated by CcpA in a glucose-independent (A) or a glucose-dependent way (B). Categories indicated are: A, Similar to unknown proteins/no similarity; B, Metabolism of carbohydrates and related molecules; C, Transport/binding proteins and lipoproteins; D, Metabolism of amino acids and related molecules; E, Adaption to atypical conditions; F, Pathogenic factors; G, Protein synthesis; H, Metabolism of nucleotides and nucleic acids; I, Metabolism of coenzymes and prosthetic groups; J, Membrane bioenergetics; K, RNA synthesis; L, Metabolism of lipids; M, Miscellaneous; N, Cell wall; O, Detoxification; P, Sensors; Q, Cell division; R, DNA replication; S, Protein folding; T, DNA recombination; U, DNA recombination.

Genes showing an altered expression in the Δ*ccpA *mutant compared to the wild-type when growing in LB alone, without glucose addition, are listed in Additional files 1: Genes with lower expression in wild-type versus Δ*ccpA *mutant, and 2: Genes with higher expression in wild-type versus Δ*ccpA *mutant. These genes made up the largest regulatory group found in our study (226 genes). Only a minor part of this group of genes (38 out of 226) contained putative *cre*-sites in their promoter regions or were part of operons with putative *cre*-sites, suggesting that CcpA may affect the expression of the majority of these genes indirectly. Such indirect effects may reflect differences in the generation of metabolites due to *ccpA *inactivation, which might serve as cofactors for the regulation of further genes, and/or to a CcpA-dependent control of regulatory proteins or RNAs. Our findings suggest that glucose-independent effects due to CcpA might play a particularly important role in *S. aureus*. For a better understanding, the genes of this category were grouped into functional classes (Fig. 3A). While unknown proteins represented the largest group (61 genes), this group was followed by proteins of carbon metabolism (26 genes), transport/binding proteins and lipoproteins (25 genes), and proteins of amino acid metabolism (19 genes).

#### Glucose-dependent, CcpA-dependent genes

All genes found to be subject to regulation by glucose in a CcpA-dependent way are depicted in the Additional files 3: CcpA dependent down-regulation by glucose, and 4: CcpA-dependent up-regulation by glucose. For consistency reasons, a few genes which were not meeting the arbitrary threshold, such as SA0605 or SA0299 (indicated by a paragraph), were included, since these genes are part of putative operons and showed a tendency towards regulation. As before, only a minor part of the affected genes/operons (48 out of 155) contained putative *cre*-sites in their promoter regions, indicating a direct control by CcpA, while the majority of genes seemed to be controlled by CcpA in a way that did not involve the interaction with an apparent *cre*-site.

Grouping the regulated genes into functional categories according to the DOGAN annotation [26] and/or KEGG database [27] showed that unknown proteins represented again the largest regulated category (39 genes), followed by transport/binding proteins and lipoproteins (22 genes), metabolism of amino acids (19 genes), and metabolism of carbohydrates (17 genes) (Fig. 3B).

#### CcpA-independent regulation by glucose

We found a small group of genes, encoding the 6-phospho-betaglucosidase, the putative ascorbate transport- and the lactose-operon, to be regulated by glucose in an apparently CcpA-independent way (Table 2). The lactose operon, reported to be controlled by catabolite repression [28] requires intracellular galactose-6-phosphate for induction [29]. The lack of specific inducer under the conditions used here may have obscured the CcpA-dependent regulatory effects on the *lac*- and other operons, or mechanisms accounting for CcpA-independent catabolite control may be active [9]. Again, the table includes a few genes not meeting the arbitrary threshold (indicated by a paragraph), which were nevertheless listed, since they are likely to form part of putative operons and showed a tendency towards regulation that was consistent with the other member(s) of these operons.

**Table 2:** Genes/operons with CcpA-independent regulation by glucose

ID			Product^a^	wt	mut
				
N315	Newman	common		+/-^b^	+/-^b^
**Down-regulated by glucose**					
SA0256	NWMN_0200	*bglA*	6-phospho-beta-glucosidase	0.5	0.5
					
SA0318	NWMN_0322		ascorbate-specific PTS system enzyme IIC	0.1	0.3
SA0319	NWMN_0323		similar to PTS system component	0.2	0.2
SA0320	NWMN_0324		similar to PTS transport system IIA component	0.2	0.2
SA0321	NWMN_0325		similar to PTS multidomain regulator	0.3	0.2
					
SA1991	NWMN_2093	*lacG*	6-phospho-beta-galactosidase	0.5	0.5
SA1992	NWMN_2094	*lacE*	PTS system, lactose-specific IIBC component	0.5	0.4
SA1993	NWMN_2095	*lacF*	PTS system, lactose-specific IIA component	0.4	0.4
SA1994	NWMN_2096	*lacD*	tagatose-1,6-diphosphate aldolase	0.5	0.4
SA1995	NWMN_2097	*lacC*	tagatose-6-phosphate kinase	0.6^§^	0.6^§^
SA1996	NWMN_2098	*lacB*	galactose-6-phosphate isomerase LacB subunit	0.5	0.4
SA1997	NWMN_2099	*lacA*	galactose-6-phosphate isomerase LacA subunit	0.6^§^	0.5

^a ^Cellular main roles are in accordance with the N315 annotation of the DOGAN website [26] and/or the KEGG website [27].

^b ^Comparison of gene expression with (+) and without (-) glucose, genes with a +/- glucose ratio of ≤ 0.5 or ≥2 in the wild-type were considered to be regulated

^§ ^Genes with regulation above threshold, which were included in the list because they were part of a putative operon.

#### Glucose-dependent genes regulated by CcpA and additional factors

One group of genes showed markedly different regulatory patterns upon glucose addition (Table 3). These patterns might reflect the interplay of two or several regulators acting on the genes/operons, indicating the presence of further glucose-responsive regulatory elements in addition to CcpA. One pattern was characterized by a parallel up- or down-regulation by glucose in wild-type and mutant, but with different ratios, exemplified by *trePCR *and *alsDS*. Another set of genes (i.e. *pstB *or *mtlF*, SA1218-1221, and SA2321) showed a divergent glucose-regulation in wild-type and mutant. A third set, represented by the *gntRKP *operon, the *ribHABD *operon, SA1961 and SA2434-SA2435, differed in expression in response to glucose in the mutant but not in the wild-type.

**Table 3:** Glucose-dependent genes regulated by CcpA and additional factors^1^

ID			Product^a^	wt	mut
				
N315	Newman	common		+/-^b^	+/-^b^
SA0432	NWMN_0438	*treP*	PTS system, trehalose-specific IIBC component	0.5	0.2
SA0433	NWNM_0439	*treC*	alpha-phosphotrehalose	0.7	0.3
SA0434	NWNM_0440	*treR*	trehalose operon repressor	0.7	0.3
					
SA1218	NWNM_1297	*pstB*	phosphate ABC transporter, ATP-binding protein (PstB)	0.5	2.6
					
SA1219	NWNM_1298		similar to phosphate ABC transporter	0.4	2.7
SA1220	NWNM_1299		similar to phosphate ABC transporter	0.3	3.7
SA1221	NWNM_1300	*pstS*	thioredoxine reductase	0.1	3.6
					
SA1586	NWNM_1659	*ribH*	6,7-dimethyl-8-ribityllumazine synthase	0.6	2.2
SA1587	NWNM_1660	*ribA*	riboflavin biosynthesis protein	0.6	1.8
SA1588	NWNM_1661	*ribB*	riboflavin synthase alpha chain	0.7	2.0
SA1589	NWNM_1662	*ribD*	riboflavin specific deaminase	0.7	2.0
					
SA1960	NWNM_2057	*mtlF*	PTS system, mannitol specific IIBC component	6.4	0.2
					
SA1961	NWNM_2058		similar to transcription antiterminator BglG family	0.9	0.4
					
SA2007	NWNM_2110	*alsD*	alpha-acetolactate decarboxylase	9.1	2.7
SA2008	NWNM_2111	*alsS*	alpha-acetolactate synthase	9.1	3.1
					
SA2293	NWNM_2401	*gntP*	gluconate permease	0.7	2.5
SA2294	NWNM_2402	*gntK*	gluconate kinase	1.6	3.7
*SA2295	NWNM_2403	*gntR*	gluconate operon transcriptional repressor	1.5	3.2
					
SA2321	NWMN_2432		hypothetical protein	0.1	2.5
SA2434	NWNM_2540		PTS system, fructose-specific IIABC component	1.2	0.4
SA2435	NWNM_2541	*pmi*	mannose-6-phosphate isomerase	1.2	0.4

^1^Genes with parallel glucose-mediated regulation in wild-type and mutant but with different ratios, genes with divergent glucose-mediated regulation in wild-type and mutant, and genes with glucose-mediated regulation in the mutant but not in the wild-type

^a ^Cellular main roles are in accordance with the N315 annotation of the DOGAN website [26]and/or the KEGG website [27].

^b ^Comparison of gene expression with (+) and without (-) glucose, genes with a +/- ratio of ≤ 0.5 or ≥2 in the wild-type and the mutant were considered to be regulated)

* Genes containing putative *cre*-sites

### Metabolic pathways under the control of CcpA

**Figure 4:**
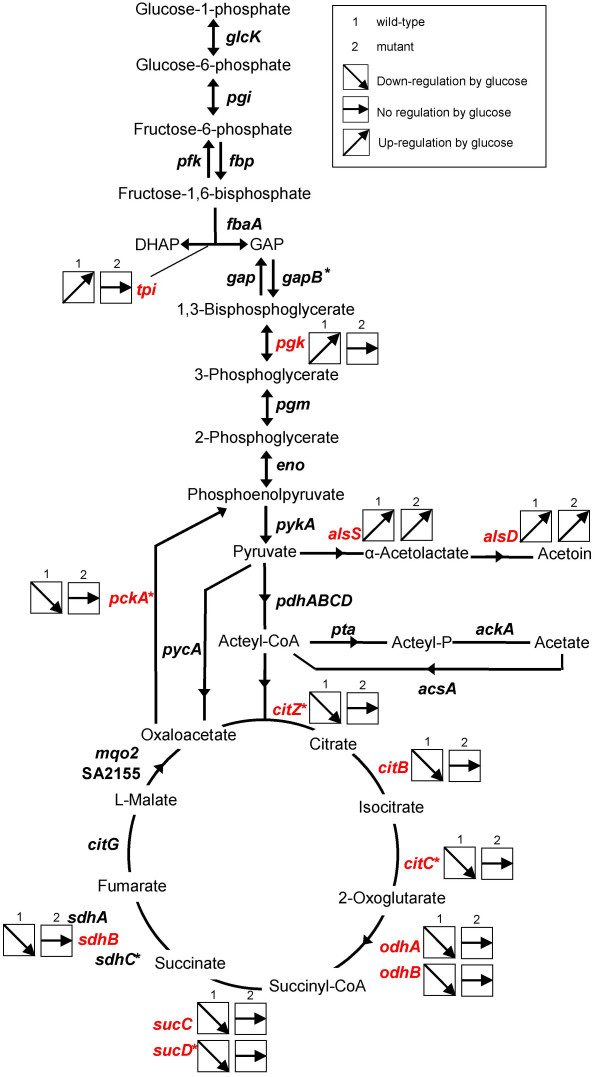
**Overview on CcpA- and glucose-dependent genes of glycolysis, gluconeogenesis and TCA cycle**. Assignment of genes coding for enzymes of glycolysis, gluconeogenesis and the TCA cycle which are regulated by CcpA. *ackA*, acetate kinase;*acsA*, acetyl-CoA synthetase; *citB*, aconitate hydratase; *citC*, citrate dehydrogenase; *citG*, fumarate hydratase; *citZ*, citrate synthase; *eno*, enolase; *fbpA*, fructose-bisphosphate aldolase; *fbp*, fructose-1,6-bisphosphatase; *gap*, glyceraldehyde-3-phosphate dehydrogenase; *gapB*, glyceraldehyde-3-phosphate dehydrogenase; *glcK*, glucokinase; *mqo2*, malate:quinone-oxidoreductase; *odhA*, 2-oxoglutarate dehydrogenase component E1; *odhB*, 2-oxoglutarate dehydrogenase component E2; *pckA*, phosphoenolpyruvate carboxykinase; *pdhABCD*, pyruvate dehydrogenase; *pfk*, phosphofructokinase; *pgi*, glucose-6-phosphate isomerase; *pgk*, phosphoglycerate kinase; *pgm*, phosphoglycerate mutase; *pycA*, pyruvate carboxylase; *pykA*, pyruvate kinase; SA2155, malate:quinone-oxidoreductase; *sdhA*, succinate dehydrogenase; *sucC*, succinyl-CoA synthetase, beta subunit; *sucD*, succinyl-CoA synthetase, alpha subunit; *tpi*, triose-3-phosphate isomerase. *, genes with putative *cre*-sites; red, regulated genes.

In *S. aureus*, glucose is mainly catabolized to pyruvate via glycolysis [30] (Fig. 4). The enzymes catalyzing the central parts of glycolysis of *S. aureus *are encoded by five genes: a glyceraldehyde-3-phosphate dehydrogenase (*gap*), phosphoglycerate kinase (*pgk*), triosephosphate isomerase (*tpi*), phosphoglyceromutase (*pgm*), and enolase (*eno*). We found that in the presence of glucose, only *tpi *and *pgk *were up-regulated by a factor of more than two in a CcpA-dependent manner (Fig. 4, Additional file 4: CcpA-dependent up-regulation by glucose). The absence of putative *cre*-sites indicated indirect control by CcpA. The other glycolytic genes also tended to show an up-regulation in transcription in response to glucose, however, below the threshold-level, and this tendency was also observed for the mutant (see Additional file 4: CcpA-dependent up-regulation by glucose).

Our microarrays confirmed previous findings [24,31], reporting a glucose-induced CcpA-mediated repression of PEP carboxykinase (*pckA*) (Fig. 4, Additional file 3: CcpA-dependent down-regulation by glucose), which is involved in gluconeogenesis. The presence of a putative *cre*-site in the promoter region of this gene indicated a direct regulation by CcpA [24,31], which contrasts with findings made in *B. subtilis*, where *pckA *was shown to be under indirect control of CcpA [32].

The pentose phosphate pathway, an alternative glucose degradation pathway in *S. aureus *[30], provides the cell with NADPH and precursors for biomass, which are needed in many anabolic reactions. *gntRKP *was the only operon of the pentose phosphate pathway we found to be regulated at least partially by CcpA (Table 3).

When glucose is depleted from the medium, *S. aureus *reintroduces products of carbon overflow, such as acetate or acetoin, into central metabolism [33,34]. The genes for acetolactate synthase (*alsS*) and acetolactate decarboxylase (*alsD*), both involved in acetoin production, were up-regulated by glucose (Table 3). Although up-regulation was found in wild-type and Δ*ccpA *mutant, it was three times higher in the wild-type, indicating a substantial contribution of CcpA in *alsD *and *alsS *transcription in response to glucose. While the amount of acetate in the medium increased upon glucose addition in both, wild-type and mutant (Fig. 1), we neither observed an increase in transcription of genes encoding proteins being involved in acetate formation (i.e. phosphotransacetylase [*pta*] and acetate kinase [*ackA*]), nor of genes with products responsible for acetate and acetoin utilization (i.e. acetyl-CoA synthetase [*acsA*], acetoin dehydrogenase [*acuA*], and the acetoin utilization protein [*acuC*]).

In the presence of glucose, CcpA repressed several genes of the TCA cycle, including aconitate hydratase (*citB*), isocitrate dehydrogenase (*citC*), and citrate synthase (*citZ*), confirming previous findings [23]. Also succinate dehydrogenase (*sdhB*), succinyl-CoA synthetase (*sucCD*), and 2-oxoglutarate dehydrogenase (*odhAB*) were repressed by glucose in a CcpA-dependent manner (Fig. 4, Additional file 3: CcpA-dependent down-regulation by glucose). The majority of promoter regions of these genes contained a putative *cre*-site (see Additional file 3: CcpA-dependent down-regulation by glucose), indicating that the TCA cycle is under direct control of CcpA.

The *pdhABCD *operon, coding for the pyruvate dehydrogenase complex, which links glycolysis to the TCA cycle by converting pyruvate to acetyl-CoA, was not found to be regulated by CcpA in *S. aureus*.

*S. aureus *is able to use amino acids as secondary carbon sources. However, this is not necessary in the presence of high amounts of glucose. Accordingly, we found that several genes coding for enzymes of amino acid degradation (*rocA, arg, rocD, glnA, hutI, hutU, aldA, ald, gudB*, SA1365, SA1366, SA1367) were repressed by glucose in a CcpA-dependent fashion (see Additional file 3: CcpA-dependent down-regulation by glucose). The genes coding for alanine dehydrogenase (*ald*), aldehyde dehydrogenase (*aldA*), arginase (*arg*), and delta-1-pyrroline-5-carboxylate dehydrogenase (*rocA*) contained putative *cre*-sites in their promoter regions (see Additional file 3: CcpA-dependent down-regulation by glucose) and might therefore be under the direct control of CcpA. According to our Northern blot findings and previously published microarray data [35], *gudB*, encoding glutamate dehydrogenase, and *rocD*, encoding ornithine aminotransferase, seemed to be co-transcribed. Interestingly, this operon contains three putative *cre*-sites (see Additional file 3: CcpA-dependent down-regulation by glucose), suggesting a complex transcriptional regulation by CcpA, which could be confirmed by our Northern blot analyses, showing that *rocD/gudB*-transcription is largely affected by CcpA in response to glucose. Similarly, *aldA, arg*, and *rocA *transcription patterns determined by Northern analyses showed the same tendency as our microarray data (Fig. 2).

Table 4 shows genes coding for transporters or lipoproteins which were regulated by glucose in a CcpA-dependent manner or which were partially controlled by CcpA. Seven of these genes contained putative *cre*-sites in their promoter regions, or as in the case of SA0186, SA0302, and *gntP*, belonged to an operon which contained a putative *cre*-site and were probably under the direct control of CcpA. The up-regulation of the glucose uptake protein homologue (SA2053) may contribute to the rapid glucose consumption observed in the wild-type (Fig. 1). Many putative non-sugar-transporters were found to be regulated by CcpA: Amongst them, the *opu*-operon, which is preceded by a putative *cre*-site and consists of *opuCA-opuCB-opuCC-opuCD*, coding for a glycine-betaine/carnitine/choline ABC transporter, acting in osmoprotection [36], was up-regulated by glucose. Interestingly, the same operon is also up-regulated in *femAB *mutants, due to a secondary effect compensating for an impaired cell envelope [37]. *S. aureus *possesses two systems involved in osmoprotection [36], the second system encoded by the *opuD *gene did not appear to be regulated by CcpA.

**Table 4:** CcpA-dependent genes coding for transport/binding proteins and lipoproteins regulated by glucose

ID			Product^a^	wt	mut
				
N315	Newman	common		+/-^b^	+/-^b^
**Down-regulated by glucose**					
SA0100	NWMN_0049		similar to Na+ P_i_-cotransporter	0.2	1.7
					
*SA0186	NWNM_0136		sucrose-specific PTS tranporter IIBC component protein	0.4	1.2
					
*SA0302	NWNM_0255		probable pyrimidine nucleoside transport protein	0.4	1.8
					
SA1848	NWNM_1950	*nrgA*	probable ammonium transporter	0.4	0.8
					
SA2226	NWNM_2337		similar to D-serine/D-alanine/glycine transporter	0.2	0.9
SA2227	NWNM_2337		amino acid ABC transporter homologue	0.1	0.9
					
**Up-regulated by glucose**					
SA0166	NWNM_0116		similar to nitrate transporter	2.8	1.1
SA0167	NWNM_0117		similar to membrane lipoprotein SrpL	2.8	1.6
SA0168	NWNM_0118		similar to probable permease of ABC transporter	2.3	1.1
					
SA0214	NWMN_0158	*uhpT*	hexose phosphate transport protein	2.1	1.1
					
SA0335	NWMN_0340		twin-arginine translocation protein TatA	2.2	1.4
					
SA0374	NWNM_0379	*pbuX*	xanthine permease	7.2	1.1
					
*SA0655	NWNM_0669	*fruA*	fructose specific permease	2.4	1.3
					
SA0769	NWNM_0780		D-methionine transport system ATP-binding protein	2.4	0.8
SA0770	NWNM_0781		D-methionine transport system permease	2.4	1.0
					
SA1270	NWNM_1347		similar to amino acid permease	2.0	1.1
					
SA2053	NWNM_2158		glucose uptake protein homologue	2.5	1.2
SA2234	NWMN_2344	*opuCD*	probable glycine betaine/carnitine/choline ABC transporter (membrane part) OpuCD	1.6	1.2
SA2235	NWMN_2345	*opuCC*	glycine betaine/carnitine/choline ABC transporter (osmoprotection) OpuCC	1.9	1.2
SA2236	NWMN_2346	*opuCB*	probable glycine betaine/carnitine/choline ABC transporter (membrane part) OpuCB	1.9	1.1
*SA2237	NWMN_2347	*opuCA*	glycine betaine/carnitine/choline ABC transporter (ATP-binding) OpuCA	2.6	1.0
					
SA2239	NWNM_2349		similar to amino acid transporter	2.2	1.1
					
SA2443	NWMN_2549		similar to accessory secretory protein Asp3	2.0	1.2
SA2444	NWMN_2550		similar to accessory secretory protein Asp2	2.3	1.3
					
**Partially controlled by CcpA**					
SA0432	NWMN_0438	*treP*	PTS system, trehalose-specific IIBC component	0.5	0.2
					
SA1218	NWNM_1297	*pstB*	phosphate ABC transporter, ATP-binding protein (PstB)	0.5	2.6
SA1219	NWNM_1298		similar to phosphate ABC transporter	0.4	2.7
SA1220	NWNM_1299		similar to phosphate ABC transporter	0.3	3.7
					
SA1960	NWNM_2057	*mtlF*	PTS system, mannitol specific IIBC component	6.4	0.2
					
*SA2293	NWNM_2401	*gntP*	gluconate permease	0.7	2.5
					
SA2434	NWNM_2540		PTS system, fructose-specific IIABC component	1.2	0.4

^a ^Cellular main roles are in accordance with the N315 annotation of the DOGAN website [26] and/or the KEGG website [27].

^b^Comparison of gene expression with (+) and without (-) glucose, genes with a +/- glucose ratio of ≤ 0.5 or ≥2 in the wild-type were considered to be regulated

*Genes containing putative *cre*-sites

### Selected CcpA-affected genes involved in virulence, pathogenicity, stress response and resistance

**Figure 5:**
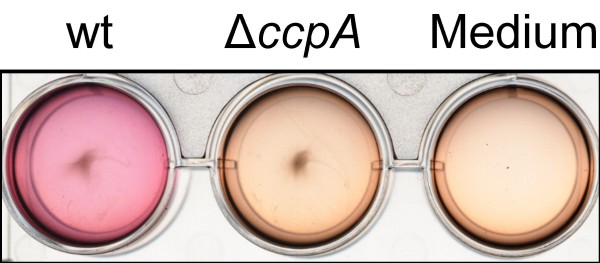
**Urease production**. Urease production in urea-containing medium. The increase in pH resulting from the cleavage of urea is indicated by a purple colour. wt, strain Newman; Δ*ccpA*, strain Newman Δ*ccpA*.

Urease is considered to be a virulence factor contributing to pathogenesis in many bacteria [38]. It hydrolyses urea into ammonia and carbon dioxide, supplying nitrogen and helping to maintain the pH stable by the formation of ammonium, allowing the adaptation to environmental changes. We noticed that irrespective of whether glucose was present in the medium or not, the urease-operon expression was higher in the wild-type than in the Δ*ccpA *mutant (see Additional file 2: Genes with higher expression in wild-type versus Δ*ccpA *mutant). Urease activity assays confirmed the transcriptional findings by showing an increased urease production by the wild-type strain in urea-containing medium compared to the Δ*ccpA *mutant (Fig. 5).

We previously observed a CcpA-dependent down-regulation of the protein A encoding gene *spa *in response to glucose [24], which was confirmed here by our transcriptional analyses (Table 5). Moreover, further genes known or thought to encode proteins being involved in immunomodulating processes, such as the immunodominant antigen A (IsaA), the staphylococcal secretory antigen SsaA and its homologue SA2353, and the *eap *domain homologue SA0841 where found to be down-regulated by glucose in the wild-type as well (Table 5). Similarly, the *atl *gene, coding for the bifunctional autolysin, important in primary attachment to glass and polystyrene surfaces [39] and reduced in intermediate glycopeptide resistant strains [40], was down-regulated by glucose in the wild-type strain. This is partially in contrast to previous findings, in which we observed a trend towards stronger *atl *expression in glucose containing TSB medium in the wild-type in comparison to a Δ*ccpA *mutant [23]. However, growth conditions and strains differed between these two studies.

**Table 5:** Regulators and factors involved in virulence and/or resistance subject to regulation by CcpA and glucose

ID			Product^a^	wt	mut
				
N315	Newman	common		+/-^b^	+/-^b^
*Glucose-dependent regulation by CcpA*					
**Down-regulated by glucose**					
*SA0107	NWNM_0055	*spa*	immunoglobulin G binding protein A precursor	0.2	1.1
					
SA0620	NWNM_0634		secretory antigen SsaA homologue	0.4	0.9
					
SA0841	NWNM_0851		similar to cell surface protein Map-w	0.4	0.9
					
SA0905	NWNM_0922	*atl*	autolysin (N-acetylmuramyl-L-alanine amidase and endo-b-N-acetylglucosaminidase)	0.4	1.1
					
SA2353	NWNM_2466		similar to secretory antigen precursor SsaA	0.5	1.0
					
SA2356	NWNM_2469	*isaA*	immunodominant antigen A	0.4	0.8
					
**Up-regulated by glucose**					
SA1010	NWNM_1076		similar to exotoxin 4	2.3	0.6
					
SA1700	NWNM_1822	*vraR*	two-component response regulator	2.2	0.8
SA1701	NWNM_1823	*vraS*	two-component sensor histidine kinase	2.5	0.7
					
SA1869	NWNM_1970	*sigB*	sigma factor B	1.7	1.0
SA1870	NWNM_1971	*rsbW*	anti-sigmaB factor	2.2	1.1
SA1871	NWNM_1972	*rsbV*	anti-sigmaB factor antagonist	1.3	0.9
SA1872	NWNM_1973	*rsbU*	sigmaB regulation protein RsbU	0.9	0.7
					
SA2290	NWNM_2397	*fnbB*	fibronectin-binding protein homologue	2.6	0.9
					
*SA2329	NWNM_2440	*cidA*	murein hydrolase regulator	3.5	1.4

^a ^Cellular main roles are in accordance with the N315 annotation of the DOGAN website [26] and/or the KEGG website [27].

^b ^Comparison of gene expression with (+) and without (-) glucose, genes with a +/- glucose ratio of ≤ 0.5 or ≥2 in the wild-type were considered to be regulated

^c ^Comparison of gene expression of wild-type (wt) and ΔccpA mutant (mut) at OD600 1 (T0) and 30 min later (T30). genes with a wt/mut ratio of ≤ 0.5 or ≥2 were considered to be regulated.

* Genes containing putative *cre*-sites

The genes coding for the two-component-system VraSR were found to be up-regulated by glucose in a CcpA-dependent manner. This system was reported to regulate the so-called cell wall stress stimulon, a set of genes that is induced in the presence of cell wall damaging agents [41]. Indeed, some of the genes, which were reported to belong to the cell wall stress stimulon of strain Newman [42] were found to be regulated by glucose in a CcpA-dependent manner as well. However, there was no specific correlation between up- and down-regulation in response to glucose and vancomycin.

Surprisingly, *rsbW*, coding for the anti-σ^b ^factor, which forms part of a polycistronic transcript that includes at least the genes *rsbUVW *and *sigB *[43], was found to be up-regulated two-fold by glucose in the wild-type in a CcpA-dependent manner, while none of the other co-transcribed genes of the *sigB *operon showed changes in expression that were above the threshold (Table 5). Interestingly, similar findings have been made by others as well [44], indicating that the *rsbUVW-sigB *transcripts might be subject to post-transcriptional processes or that further, yet unidentified promoters within the *sigB *operon might exist, which would lead to increased *rsbW *transcription.

The gene coding for the fibronectin binding protein B (*fnbB*), was up-regulated in the wild-type by glucose. Although this protein is truncated and not functional in strain Newman [45,46], it might be regulated by CcpA in strains where it is functional, suggesting, that CcpA may affect also adherence and host cell invasion [47].

The microarray data confirmed previously published data, in which we found *cidA *transcription to be higher in the wild-type than in the Δ*ccpA *mutant in the presence of glucose [23]. CidA, controlling cell lysis and the release of extracellular DNA (eDNA), was shown to contribute to biofilm formation [48], which is strongly induced in the presence of glucose [23].

### Differential analysis of the cytoplasmic proteome of wild-type and Δ*ccpA *mutant

To complement our transcriptional data, we also compared the cytoplasmic proteome of the wild-type (Newman) and its isogenic Δ*ccpA *mutant grown in buffered LB medium in the presence and absence of glucose. The protein patterns under both conditions were compared and proteins, whose amounts were affected by the addition of glucose, were identified by mass spectrometry.

**Figure 6:**
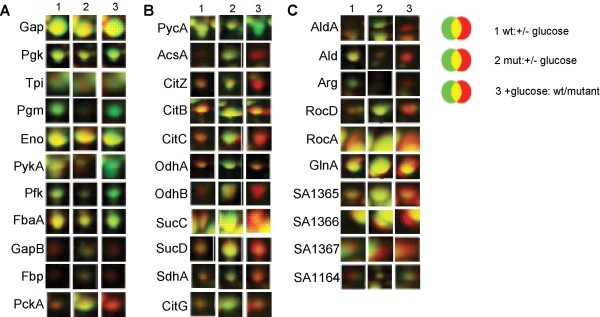
**Amounts of selected proteins representing different branches of metabolism**. A, glycolysis/gluconeogenesis; B, TCA cycle; and C, amino acid degradation. Differential protein amounts 1 h after addition of glucose to exponentially growing cells are shown. The protein levels in the wild-type (1) and mutant (2) in the presence of glucose (green) were compared with the protein levels in the absence of glucose (red). In addition, the protein levels in the presence of glucose (3) in the wild-type (green) were compared to those in the mutant (red).

In the presence of glucose, increased amounts of components of the glycolytic pathway such as Pfk, Tpi, Pgk, Pgm, Eno, Gap and PykA were observed in the wild-type (Fig. 6A). Proteins of gluconeogenesis, namely the gluconeogenic glyceraldehyde-3P-dehydrogenase (GapB), fructose bisphosphatase (Fbp), and PEP carboxykinase (PckA) were present at lower levels in the presence of glucose in the wild-type, while in the mutant, the amounts were not altered in response to glucose (Fig. 6A). Also the production of acetyl-CoA-synthetase (AcsA) was clearly down-regulated by glucose in a CcpA-dependent manner (Fig. 6B).

In line with the transcriptional findings, the level of TCA cycle enzymes detetctable on 2D gels (CitZ, CitB, CitC, OdhA/B, SucC, SucD, SdhA, CitG) was found to be clearly reduced in the wild-type after addition of glucose (Fig. 6B).

*S. aureus *encodes two malate:quinone oxidoreductases: Mqo2 and SA2155. While the amount of Mqo2 was not affected by glucose, the amount of SA2155 as the other TCA cycle enzymes was strongly reduced (data not shown). Interestingly, pyruvate carboxylase (PycA), which is needed to replenish the pool of TCA intermediates, was found to be increased by glucose in the wild-type but not in the mutant (Fig. 6B).

In contrast to *B. subtilis *[32,49], the expression of AckA and Pta, being involved in the overflow metabolism, was not affected by CcpA and/or glucose (data not shown). Neither could we detect an effect of CcpA or glucose on the amount of the pentose phosphate pathway-enzymes, suggesting that considerable differences between *S. aureus *and *B. subtilis *exist in the CcpA-dependent regulation of the pentose phosphate pathway and carbon overflow [32].

In accordance with our microarray data, several enzymes of amino acid degradation (RocA, RocD, GudB, Ald, AldA, GlnA, and Dho) were repressed by glucose in a CcpA-dependent manner (Fig. 6C).

## Conclusion

The catabolite control protein A is likely to regulate transcription either directly, by binding to catabolite responsive elements (*cre*-sites), or indirectly by affecting the expression of regulatory molecules which in turn alter the transcription of their target genes. We previously observed that CcpA of *S. aureus *affects the expression of *RNAIII *[24], the effector molecule of the *agr *locus, and one of the major regulators of virulence determinant production of this organism [50]. Aiming at the identification of genes that are directly affected by CcpA in response to glucose, we chose an experimental setup in which we gave a glucose-impulse to exponentially growing wild-type and Δ*ccpA *mutant cells and analyzed the effect 30 min (transcriptome) and 60 min (proteome) after the glucose addition. While this strategy was likely to reduce putative side-effects, such as the CcpA-dependent regulation of *RNAIII *expression or pH-effects, which in turn would have a significant effect on the transcriptional and proteomic profiles, it also limited this study to detect only short-term effects of CcpA in response to glucose. It did neither allow the identification of the glucose-induced long-term effects of CcpA on the transcriptome, nor the effect of CcpA on the transcription of genes that are predominantly expressed during the later stages of growth. Thus, one particular consequence of our strategy might have been the overrepresentation of genes/operons found to be affected by the *ccpA *inactivation in the absence of glucose, which contrasts with findings made in *B. subtilis *[50], where the glucose-induced effect of CcpA on the transcriptome clearly exceeded the number of genes that were affected by CcpA in a glucose-independent manner [50]. It is feasible that the number of genes being affected by CcpA in *S. aureus *in response to glucose would be higher if a later time-point for the glucose-impulse and/or the analysis would have been chosen, or if appropriate inducers of regulated operons had been present under the conditions analyzed. Another surprising observation that we encountered was the high degree of genes found to be affected by CcpA in a glucose-dependent manner that lacked an apparent *cre*-site (107 out of 155). This suggests to us that the *S. aureus *CcpA might regulate transcription on a significant level in a way that does not require binding to *cre*. Changes in the metabolite content and secondary regulatory elements in the Δ*ccpA *mutant may be possible explanations. Further, CcpA might bind to a *cre *consensus, which is composed much broader than the one used by us in this study for the identification of putative *cre*-sites.

In general, overall induction or repression levels of CcpA were low, showing mostly values around the threshold level of 2 and 0.5, respectively. However, inactivation of *ccpA *still leads to drastic alteration in the transcriptome and the proteome of the bacterium, affecting not only major metabolic pathways, but also resistance, virulence and biofilm formation [22-24], which are properties contributing to the adaptation to environmental stress. However, the impact of catabolite repression on staphylococcal virulence in the host can not be predicted by the *in vitro *data and needs to be assessed experimentally. Environmental conditions, carbon sources, pH etc. differ strongly upon the site of infection and underlying diseases, such as diabetes.

Although overall regulation of central carbon metabolism mediated by CcpA was found to be similar to the one in the model organism *B. subtilis*, the extent to which this control was exerted seemed to differ in some aspects between these two bacteria. CcpA regulation of *S. aureus *seemed to differ in terms of overflow metabolism from *B. subtilis*, since in addition to *alsS*, *pta *and *ackA *where found to be regulated by glucose in a CcpA-dependent way in *B. subtilis *[34,51,52], but not in *S. aureus*. Also the genes responsible for acetoin utilization (i.e. acetoin dehydrogenase [*acuA*], and the acetoin utilization protein [*acuC*]), where regulated in a CcpA-dependent manner in *B. subtilis *[53], but not in *S. aureus*. These genes may however be regulated at a later time point during growth. Another difference was the regulation of the *pdhABCD *genes, coding for pyruvate dehydrogenase, which were activated by glucose in *B. subtilis *but not in *S. aureus *[32]. Moreover, we found no CcpA-dependent regulation of glutamate synthase (*gltBD*), which catalyses the conversion of glutamate to 2-oxoglutarate, again in contrast to the findings in *B. subtilis*, in which the transcription of these genes is induced in response to glucose by CcpA [2]. Also different to *B. subtilis *was the finding that none of the genes devoted to branched-chain amino acids where induced by the presence of glucose in *S. aureus *[54-56]. However, in a transcriptome analysis over time, Lulko et *al*. [5] only observed CcpA-mediated regulation of these genes in the late-exponential growth (transition) phase in *B. subtilis*. Thus, it is possible, that also in *S. aureus *these genes might be regulated by glucose in a CcpA-dependent manner at a later growth phase.

## Methods

### Bacterial strains and growth conditions

*S. aureus *Newman [57] and its isogenic Δ*ccpA *mutant MST14 [24] were grown in LB medium buffered with 50 mM HEPES (pH 7.5) in Erlenmeyer flasks with a culture to flask volume of 1:5 under vigorous agitation at 37°C to an optical density (OD_600_) of 1.0. One half of the culture was transferred to a new Erlenmeyer flask and glucose was added to a final concentration of 10 mM, while the other half remained without glucose. Samples for microarray analysis were taken at OD_600 _of 1.0 (T0) and after 30 minutes (T30). Total RNA was extracted as previously described [58,59]. For proteome analysis cells were grown with a culture to flask volume of 1:10 under vigorous agitation until an OD_600 _of 1.0 and glucose was added to one half of the culture. To allow protein accumulation, samples were taken 60 min afterwards from both, the culture to which glucose was added, and the culture which remained without glucose.

### Microarray design and manufacturing

The microarray was manufactured by *in situ *synthesis of 10'807 different oligonucleotide probes of 60 nucleotides length (Agilent, Palo Alto, CA, USA), selected as previously described [60]. It covers approximately 99% of all ORFs annotated in strains N315 and Mu50 [61], MW2 [62] and COL [63] including their respective plasmids [59]. Extensive experimental validation of this array has been described previously, using CGH, mapping of deletion, specific PCR and quantitative RT-PCR [60,64].

### Expression microarrays

DNA-free total RNA was obtained after DNase treatment on RNeasy columns (Qiagen) [58,59]. The absence of remaining DNA traces was evaluated by quantitative PCR (SDS 7700; Applied Biosystems, Framing-ham, MA) with assays specific for 16s rRNA [58,59]. Batches of 8 μg total *S. aureus *RNA were labelled by Cy-3 or Cy-5 dCTP using the SuperScript II (Invitrogen, Basel, Switzerland) following manufacturer's instructions. Labelled products were purified onto QiaQuick columns (Qiagen) and mixed with 250 μl Agilent hybridization buffer, and then hybridized at a temperature of 60°C for 17 h in a dedicated hybridization oven (Robbins Scientific, Sunnyvale, CA, USA). Slides were washed with Agilent proprietary buffers, dried under nitrogen flow, and scanned (Agilent, Palo Alto, CA, USA) using 100% PMT power for both wavelengths.

### Microarray analysis

Fluorescence intensities were extracted using the Feature extraction™ software (Agilent, version 8). Local background-subtracted signals were corrected for unequal dye incorporation or unequal load of labelled product. The algorithm consisted of a rank consistency filter and a curve fit using the default LOWESS (locally weighted linear regression) method. Data consisting of two independent biological experiments were analyzed using GeneSpring 7.3 (Agilent). An additional filter was used to exclude irrelevant values. Background noise of each experiment was evaluated by computing the standard deviation of negative control intensities. Features whose intensities were smaller than the standard deviation value of the negative controls in all the measurements were considered as inefficient hybridization and discarded from further analysis [64]. Fluorescence values for genes mapped by 2 probes or more were averaged. Statistical significance of differentially expressed genes was identified by variance analysis (ANOVA) [59,65], performed using GeneSpring, including the Benjamini and Hochberg false discovery rate correction (5%). A gene was considered to be regulated by glucose and/or CcpA if transcription was induced or repressed at least two fold. Microarray data were submitted to the GEO database with accession numbers GPL3931 and GSE12614 for the complete experimental data set.

### Evaluation of the microarray data

Several classes of effects could be observed. Genes, which showed differences in total transcriptome between wild-type and mutant in the absence of glucose at both time points, e.g. OD_600 _of 1 (T0) and after 30 min (T30), were considered to be CcpA-dependent, but glucose-independent. When a difference was only observed at one of the two time points or the gene was up-regulated at one and down-regulated at the other time point, it was assumed to have fluctuating expression patterns and was not considered in this study. Genes with a differential expression upon glucose addition in the wild-type but not in the Δ*ccpA *mutant were considered to be strictly CcpA-dependent. Changes occurring in parallel in the wild-type and the mutant were considered to be due to glucose, but CcpA-independent. A last group comprised genes, which were found to be affected in their expression in response to glucose in both wild-type and mutant, but with differing ratios, or genes, which showed no regulation in the wild-type, but regulation in the mutant upon glucose addition. This group of genes was considered to be controlled by CcpA and other regulatory proteins at the same time.

For a better interpretation, the organization of genes in putative operons was deduced from the transcriptional profiles of adjacent genes over time according to previous microarrays [35] and by searching for putative terminator sequences using TransTerm [66].

### Northern blot analyses

For Northern blot analysis cells were centrifuged for 2 min at 12,000 × g and cell-sediments snap-frozen in liquid nitrogen. RNA isolation and Northern blotting were performed as described earlier [67]. Primer-pairs are shown in Additional file 5: Primers used for the construction of DIG-labelled DNA probes. All Northern blot analyses were performed at least twice on independently isolated RNA samples.

### Identification of putative S. aureus cre-sites

Regulated genes were analyzed by screening for putative *cre*-sites using the *B. subtilis *consensus sequence (WWTGNAARCGNWWWCAWW) suggested by Miwa et *al. *2000 [7]. Being aware that diverse *cre*-site consensi have been published [7,8,68-70], we allowed up to two mismatches in the staphylococcal *cre *candidates. To constrict the *cre*-sites identified, we evaluated the presence of palindromic parts.

### Preparation of cytoplasmic proteins for two-dimensional (2D) polyacrylamide gel electrophoresis (PAGE)

Cells of 40 ml culture were harvested on ice and centrifuged for 5 min at 7000 g and 4°C. Cells were washed three times with ice-cold TE (10 mM Tris, 1 mM EDTA, pH 7.5) and resuspended in 1.1 ml TE buffer. For mechanical disruption, the cell suspension was transferred to screw-cap microtubes (Sarstedt, Germany) containing 500 μl of glass beads (diameter 0.10 – 0.11 mm, Sartorius, Goettingen, Germany). Cells were disrupted by homogenization using a Ribolyser (Thermo Electron Corporation, USA) at 6.5 m/s for 35 seconds. The lysate was centrifuged for 25 min at 21'000 × *g *(4°C). In order to remove membrane fragments and insoluble proteins, the centrifugation step was repeated for 45 min at 21,000 × *g *(4°C). The protein concentration was determined using Roti Nanoquant (Roth, Germany), and the protein solution was stored at -20°C.

### Analytical and preparative 2D-PAGE

2D-PAGE was performed using the immobilized pH gradient (IPG) technique described previously [71]. In the first dimension, the protein samples (300 μg) were separated on IPG strips (GE-Healthcare, Little Chalfont, United Kingdom) in the pH range of 4 to 7. The proteins were stained with colloidal Coomassie Brillant Blue [72]. The stained gels were scanned with a light scanner with integrated transparency unit (Quatographic, Braunschweig, Germany).

### Protein identification by mass spectrometry

For identification of proteins by MALDI-TOF-MS, Coomassie stained protein spots were cut from gels using a spot cutter (Proteome WorkTM) with a picker head of 2 mm and transferred into 96-well microtiter plates. Digestion with trypsin and subsequent spotting of peptide solutions onto the MALDI targets were performed automatically in the Ettan Spot Handling Workstation (GE-Healthcare, Little Chalfont, United Kingdom) using a modified standard protocol [73]. MALDI-TOF-MS analyses of spotted peptide solutions were carried out on a Proteome-Analyzer 4700 (Applied Biosystems, Foster City, CA, USA). The spectra were recorded in a reflector mode in a mass range from 900 to 3700 Da. Automatic or manual calibration was performed as described by [73]. After calibration, the peak lists were created using the "peak to mascot" script of the 4700 ExplorerTM software. The resulting peak lists were analyzed by using the mascot search engine (Matrix Science, London, UK), GPMAW 4.1 (Lighthouse data). The annotation of *S. aureus *N315 was used for protein identification and denotation. Peptide mixtures that yielded at least twice a Mowes score of at least 50 and a sequence coverage of at least 30% were regarded as positive identifications. Proteins that failed to exceed the 30% sequence coverage cut-off were subjected to MALDI-MS/MS [73]. Database searches were performed using the Mascot search engine with the protein databases of *S. aureus *strain N315.

### Protein quantitation approaches

The 2D gel image analysis was performed with the software "Delta2D" (DECODON GmbH, Greifswald, Germany). Three different data sets were analyzed in order to screen for differences in the amount of cytoplasmic proteins identified on 2D gels.

### Detection of glucose, acetate and lactate

The concentrations of glucose, acetate and lactate in the supernatants were determined using commercially available kits (Boehringer) according to the manufacturer's instructions.

### Urease assay

McFarland 0.5-standard cell suspensions were diluted 100-fold in urea medium [74] and incubated in 12-well plates at 37° for 24 hours. In parallel, colony forming units (cfu/ml) were determined.

## Authors' contributions

KS experimentally validated the microarray data, performed computational analyses of *cre*-sites, Northern blot analyses, urease assays, contributed to the interpretation of the results, and drafted the manuscript. SM confirmed some of the Northern blot experiments and the urease assays. PF of the group of JS carried out the microarrays and performed statistical analyses. SE and CK performed the proteome analysis. MB and BBB conceived, and coordinated the study, and participated in writing the manuscript. All authors read and approved the final manuscript.

## Supplementary Material

Additional file 1 **Genes with lower expression in wild-type versus Δ*ccpA *mutant**. The table represents genes showing a lower gene expression in the wild-type than the Δ*ccpA *mutant (wt/mutant ratio ≤ 0.5). Cells were grown in LB, without glucose addition. Click here for file

Additional file 2 **Genes with higher expression in wild-type versus Δ*ccpA *mutant**. The table represents genes showing a higher gene expression in the wild-type than the Δ*ccpA *mutant (wt/mutant ratio ≥ 2.0). Cells were grown in LB, without glucose addition. Click here for file

Additional file 3 **CcpA-dependent down-regulation by glucose**. The table shows genes found to be subject to down-regulation by glucose in a CcpA-dependent manner (with/without glucose ratio of 0.5 or lower in wild-type, with/without glucose ratio of approximately 1, but below 2 in the mutant). Click here for file

Additional file 4 **CcpA-dependent up-regulation by glucose**. The table shows genes found to be subject to up-regulation by glucose in a CcpA-dependent manner (with/without glucose ratio of 2 or higher in wild-type, with/without glucose ratio of approximately 1, but below 2 in the mutant). Click here for file

Additional file 5 **Primers used for the construction of DIG-labelled DNA probes**. Click here for file
