# Supplementary material for: Effect of a glucose impulse on the CcpA regulon in Staphylococcus aureus
Source: BMC Microbiol. 2009 May 18;9:95. doi: 10.1186/1471-2180-9-95 (PMC2697999; doi:10.1186/1471-2180-9-95)
Supplement: Additional file 1 — Genes with lower expression in wild-type versus ΔccpA mutant. The table represents genes showing a lower gene expression in the wild-type than the ΔccpA mutant (wt/mutant ratio ≤ 0.5). Cells were grown in LB, without glucose addition. [file 1471-2180-9-95-S1.doc]

### Additional file 1 – Genes with lower expression in wild-type versus Δ*ccpA* mutant

| ID | |  |  | wt/mut | wt/mut |  |  |
| --- | --- | --- | --- | --- | --- | --- | --- |
| N315 | Newman | common | Product^a^ | T0b | T30b | *cre*^c^ | Position |
| SA0107 | NWMN_0055 | *spa* | immunoglobulin G binding protein A precursor | 0.4 | 0.2 | TATTAAACCGCTTTCATT | -221 TTG |
| SA0108 | NWMN_0056 | *sarH1* | staphylococcal accessory regulator A homologue | 0.4 | 0.2 |  |  |
| SA0128 | NWMN_0077 | *sodM* | superoxide dismutase | 0.6 | 0.5 |  |  |
| SA0131 | NWMN_0080 | *pnp* | purine nucleoside phosphorylase | 0.6 | 0.2 | CTTGAAAGCGCTTTTAAA | -68 ATG |
| SA0143 | NWMN_0094 | *adhE* | alcohol-acetaldehyde dehydrogenase | 0.3 | 0.3 |  |  |
| SA0183 | NWMN_0133 | *glcA* | PTS enzyme II (EC 2.7.1.69), glucose-specific, factor IIA homologue | 0.4 | 0.2 |  |  |
| SA0213 | NWMN_0157 |  | conserved hypothetical protein | 0.4 | 0.3 |  |  |
| SA0216 | NWMN_0160 |  | similar to two-component sensor histidine kinase | 0.5 | 0.4 |  |  |
| SA0218 | NWMN_0162 | *pflB* | formate acetyltransferase | 0.3 | 0.2 | TATGAAAACGTTAACATA | -42 ATG |
| SA0219 | NWMN_0163 | *pflA* | formate acetyltransferase activating enzyme | 0.3 | 0.2 | TATGAAAACGTTAACATA |  |
| SA0231 | NWMN_0175 |  | similar to flavohemoprotein | 0.5 | 0.7 | GATGTAATCGTATACAAA | -301 ATG |
| SA0299 | NWMN_0253 |  | similar to carbohydrate kinase, PfkB family | 0.3 | 0.1 | AATGTAAGCGTTTACAAC | -76 ATG |
| SA0300 | NWMN_0253 |  | truncated hypothetical protein | 0.1 | 0.1 | AATGTAAGCGTTTACAAC |  |
| SA0301 | NWMN_0254 |  | conserved hypothetical protein | 0.1 | 0.1 | AATGTAAGCGTTTACAAC |  |
| SA0302 | NWMN_0255 |  | probable pyrimidine nucleoside transport protein | 0.2 | 0.1 | AATGTAAGCGTTTACAAC |  |
| SA0304 | NWMN_0257 | *nanA* | N-acetylneuraminate lyase subunit | 0.7 | 0.3 | ATTGTAAACGCTATCATA | -48 ATG |
| SA0305 | NWMN_0258 |  | similar to glucokinase | 0.6 | 0.4 | ATTGTAAACGCTATCATA | -92 ATG |
| SA0354 | NWMN_0359 | *rpsR* | 30S ribosomal protein S18 | 0.7 | 0.5 |  |  |
| SA0374 | NWMN_0379 | *pbuX* | xanthine permease | 0.6 | 0.4 |  |  |
| SA0376 | NWMN_0381 | *guaA* | GMP synthase (glutamine-hydrolyzing) | 0.7 | 0.5 |  |  |
| SA0387 | NWMN_0394 | *set11* | exotoxin 11 (Pathogenicity island SaPIn2) | 0.2 | 0.2 | AAGTTAAGCGATAACATA | -371 ATG |
| SA0389 | NWMN_0396 | *set13* | exotoxin 13 (Pathogenicity island SaPIn2) | 0.7 | 0.5 |  |  |
| SA0390 | NWMN_0397 | *set14* | exotoxin 14 (Pathogenicity island SaPIn2) | 0.5 | 0.3 |  |  |
| SA0433 | NWMN_0439 |  | alpha-glucosidase | 0.5 | 0.2 |  |  |
| SA0434 | NWMN_0440 |  | similar to trehalose operon transcriptional repressor | 0.6 | 0.3 |  |  |
| SA0452 | NWMN_0457 | *veg* | VEG protein homologue | 0.4 | 0.4 |  |  |
| SA0479 | NWMN_0483 | *nupC* | pyrimidine nucleoside transport protein | 0.7 | 0.5 | AATGGAAGGGCTAACATC | -47 ATG |
| SA0495 | NWMN_0499 | *rplK* | 50S ribosomal protein L11 | 0.5 | 0.5 |  |  |
| SA0496 | NWMN_0500 | *rplA* | 50S ribosomal protein L1 (BL1) | 0.6 | 0.3 |  |  |
| SA0497 | NWMN_0501 | *rplJ* | 50S ribosomal protein L10 (BL5) | 0.7 | 0.5 |  |  |
| SA0562 | NWMN_0577 | *adh1* | alcohol dehydrogenase I | 0.3 | 0.4 |  |  |
| SA0598 | NWMN_0612 | *pbp4* | penicillin binding protein 4 | 0.4 | 0.6 |  |  |
| SA0642 | NWMN_0656 |  | similar to cobalamin synthesis related protein | 0.7 | 0.5 |  |  |
| SA0687 | NWMN_0701 | *nrdF* | ribonucleoside-diphosphate reductase minor subunit | 0.5 | 0.7 |  |  |
| SA0769 | NWMN_0780 |  | D-methionine transport system ATP-binding protein | 0.6 | 0.4 |  |  |
| SA0770 | NWMN_0781 |  | D-methionine transport system permease | 0.6 | 0.4 |  |  |
| SA0820 | NWMN_0830 | *glpQ* | glycerophosphoryl diester phosphodiesterase | 0.7 | 0.5 |  |  |
| SA0868 | NWMN_0880 | *nrdE* | ribonucleoside-diphosphate reductase major subunit | 0.6 | 0.4 | ATTTTAAGCGCAGTCATT | +1116 ATG |
| SA0908 | NWMN_0925 |  | conserved hypothetical protein | 0.6 | 0.5 |  |  |
| SA0923 | NWMN_0939 | *purM* | phosphoribosylformylglycinamidine cyclo-ligase PurM | 0.6 | 0.5 |  |  |
| SA0924 | NWMN_0940 | *purN* | phosphoribosylglycinamide formyltransferase | 0.5 | 0.4 |  |  |
| SA0925 | NWMN_0941 | *purH* | bifunctional purine biosynthesis protein PurH | 0.4 | 0.3 |  |  |
| SA0926 | NWMN_0942 | *purD* | phosphoribosylamine--glycine ligase PurD | 0.7 | 0.5 |  |  |
| SA0937 | NWMN_0952 | *cydA* | cytochrome D ubiquinol oxidase subunit 1 homologue | 0.4 | 0.6 | TATCATTTCGTTTCGATA | -311 TTG |
| SA0938 | NWMN_0953 | *cycB* | cytochrome D ubiquinol oxidase subunit II homologue | 0.3 | 0.5 | TATCATTTCGTTTCGATA |  |
| SA1000 | NWMN_1066 |  | similar to fibrinogen-binding protein | 0.4 | 0.7 |  |  |
| SA1001 | NWMN_1067 |  | hypothetical protein | 0.4 | 0.3 |  |  |
| SA1010 | NWMN_1076 |  | similar to exotoxin 4 | 0.3 | 0.1 |  |  |
| SA1011 | NWMN_1077 |  | similar to exotoxin 3 | 0.5 | 0.3 |  |  |
| SA1012 | NWMN_1078 | *argF* | ornithine carbamoyltransferase | 0.4 | 0.3 | TTTGTATGCGCTTACAAT | -25 TTG |
| SA1013 | NWMN_1079 |  | similar to carbamate kinase | 0.3 | 0.3 | TTTGTATGCGCTTACAAT |  |
| SA1081 | NWMN_1148 | *rpsP* | 30S ribosomal protein S16 | 0.5 | 0.4 |  |  |
| SA1099 | NWMN_1166 | *rpsB* | 30S ribosomal protein S2 | 0.7 | 0.4 |  |  |
| SA1162 | NWMN_1238 |  | hypothetical protein | 0.6 | 0.3 | TGTGAAAGCGCTTGCTTA | -10 TTG |
| SA1240 | NWMN_1321 |  | conserved hypothetical protein | 0.6 | 0.5 |  |  |
| SA1241 | NWMN_1322 |  | similar to nitric-oxide reductase | 0.6 | 0.5 |  |  |
| SA1269 | NWMN_1346 |  | Blt-like protein | 0.4 | 0.4 |  |  |
| SA1270 | NWMN_1347 |  | similar to amino acid permease | 0.2 | 0.3 |  |  |
| SA1271 | NWMN_1348 |  | threonine deaminase IlvA homologue | 0.2 | 0.3 |  |  |
| SA1272 | NWMN_1349 |  | alanine dehydrogenase | 0.1 | 0.2 | AATGATTACTTTAGCATT | +569 ATG |
| SA1301 | NWMN_1378 | *ndk* | nucleoside diphosphate kinase | 0.6 | 0.4 |  |  |
| SA1442 | NWMN_1515 |  | similar to caffeoyl-CoA O-methyltransferase | 0.4 | 0.4 |  |  |
| SA1472 | NWMN_1548 |  | conserved hypothetical protein | 0.6 | 0.5 |  |  |
| SA1473 | NWMN_1549 | *rplU* | 50S ribosomal protein L21 (BL20) | 0.6 | 0.5 |  |  |
| SA1493 | NWMN_1563 | *hemD* | uroporphyrinogen III synthase | 0.5 | 0.4 |  |  |
| SA1494 | NWMN_1564 | *hemC* | porphobilinogen deaminase | 0.5 | 0.4 |  |  |
| SA1495 | NWMN_1565 | *hemX* | HemA concentration negative effector hemX | 0.6 | 0.5 |  |  |
| SA1497 | NWMN_1567 |  | conserved hypothetical protein | 0.5 | 0.5 |  |  |
| SA1499 | NWMN_1569 | *tig* | trigger factor (prolyl isomerase) | 0.5 | 0.5 |  |  |
| SA1503 | NWMN_1573 | *rpmI* | 50S ribosomal protein L35 | 0.3 | 0.4 |  |  |
| SA1504 | NWMN_1574 | *infC* | translation initiation factor IF-3 infC | 0.3 | 0.4 |  |  |
| SA1506 | NWMN_1576 | *thrS* | threonyl-tRNA synthetase 1 | 0.2 | 0.3 |  |  |
| SA1533 | NWMN_1605 | *ackA* | acetate kinase homologue | 0.4 | 0.4 |  |  |
| SA1554 | NWMN_1626 | *acsA* | acetyl-CoA synthetase | 0.5 | 0.2 | TGTGAAAACGCTTTCTTT | -21 ATG |
| SA1555 | NWMN_1627 | *acuA* | acetoin dehydrogenase homologue | 0.5 | 0.3 | TGTGAAAACGCTTTCTTT | -130 ATG |
| SA1569 | NWMN_1641 |  | conserved hypothetical protein | 0.5 | 0.6 |  |  |
| SA1585 | NWMN_1658 |  | proline dehydrohenase homologue | 0.5 | 0.2 |  |  |
| SA1608 | NWMN_1680 | *metK* | S-adenosylmethionine synthetase | 0.5 | 0.3 | AATGTAAGCCTTTACATT | -225 ATG |
| SA1617 | NWMN_1688 |  | hypothetical protein | 0.7 | 0.5 | TTTAAAAACTTTTTCAAA | -274 ATG |
| SA1649 | NWMN_1722 |  | conserved hypothetical protein | 0.5 | 0.7 |  |  |
| SA1701 | NWMN_1823 | *vraS* | two-component sensor histidine kinase | 0.7 | 0.3 |  |  |
| SA1702 | NWMN_1824 |  | conserved hypothetical protein | 0.7 | 0.4 |  |  |
| SA1710 | NWMN_1832 |  | similar to DNA polymerase III, alpha chain PolC type | 0.4 | 0.5 |  |  |
| SA1755 | NWMN_1877 |  | hypothetical protein (Bacteriophage phiN315) | 0.4 | 0.4 |  |  |
| SA1813 | NWMN_1928 |  | similar to leukocidin chain lukM precursor | 0.5 | 0.7 |  |  |
| SA1847 | NWMN_1949 | *scrR* | sucrose operon repressor | 0.7 | 0.3 | TGTGTAACCGCTTTAAAT | -49 TTG |
| SA1849 | NWMN_1951 |  | conserved hypothetical protein | 0.5 | 0.4 |  |  |
| SA1929 | NWMN_2031 | *pyrG* | CTP synthase | 0.4 | 0.4 |  |  |
| SA1960 | NWMN_2057 | *mtlF* | PTS system, mannitol specific IIBC component | 0.6 | 0.3 | ATTGTAAACGTTTAATAA | +780 ATG |
| SA2007 | NWMN_2110 | *alsD* | similar to alpha-acetolactate decarboxylase | 0.2 | 0.5 |  |  |
| SA2008 | NWMN_2111 | *alsS* | alpha-acetolactate synthase | 0.2 | 0.6 |  |  |
| SA2022 | NWMN_2125 | *rplQ* | 50S ribosomal protein L17 (BL15) (BL21) | 0.7 | 0.3 |  |  |
| SA2023 | NWMN_2126 | *rpoA* | DNA-directed RNA polymerase alpha chain | 0.7 | 0.3 |  |  |
| SA2163 | NWMN_2275 |  | hypothetical protein | 0.5 | 0.5 |  |  |
| SA2168 | NWMN_2280 |  | hypothetical protein | 0.6 | 0.5 | ATTGTAACCGGTTCCAAA | -120 ATG |
| SA2171 | NWMN_2283 |  | hypothetical protein | 0.5 | 0.4 |  |  |
| SA2172 | NWMN_2284 | *gltT* | proton/sodium-glutamate symport protein | 0.6 | 0.5 |  |  |
| SA2173 | NWMN_2285 |  | hypothetical protein | 0.3 | 0.4 |  |  |
| SA2187 | NWMN_2299 | *nirD* | assimilatory nitrite reductase | 0.5 | 0.3 |  |  |
| SA2188 | NWMN_2300 | *nirB* | nitrite reductase | 0.5 | 0.4 |  |  |
| SA2192 | NWMN_2304 |  | hypothetical protein | 0.4 | 0.4 |  |  |
| SA2201 | NWMN_2312 |  | similar to ABC transporter, permease protein | 0.5 | 0.5 |  |  |
| SA2202 | NWMN_2313 |  | similar to ABC transporter, periplasmic amino acid-binding protein | 0.5 | 0.5 |  |  |
| SA2206 | NWMN_2317 | *sbi* | IgG-binding protein SBI | 0.4 | 0.5 |  |  |
| SA2302 | NWMN_2412 |  | similar to ABC transporter | 0.3 | 0.2 |  |  |
| SA2303 | NWMN_2413 |  | similar to membrane spanning protein | 0.5 | 0.3 |  |  |
| SA2304 | NWMN_2414 | *fbp* | fructose-bisphosphatase | 0.6 | 0.3 |  |  |
| SA2410 | NWMN_2515 | *nrdD* | anaerobic ribonucleoside-triphosphate reductase | 0.3 | 0.7 |  |  |
| SA2423 | NWMN_2529 | *clfB* | clumping factor B | 0.3 | 0.2 |  |  |
| SA2425 | NWMN_2531 | *arcC* | carbamate kinase | 0.4 | 0.6 |  |  |
| SA2432 | NWMN_2538 |  | conserved hypothetical protein | 0.4 | 0.4 |  |  |
| SA2433 | NWMN_2539 |  | similar to transcription antiterminator BglG family | 0.4 | 0.2 |  |  |
| SA2434 | NWMN_2540 |  | fructose phosphotransferase system enzyme fruA homologue | 0.2 | 0.1 |  |  |
| SA2435 | NWMN_2541 | *pmi* | mannose-6-phosphate isomerase | 0.1 | 0.1 |  |  |
| SA2480 | NWMN_2586 | *drp35* | Drp35 | 0.7 | 0.1 | AATGATAACGGTTTCATC | -71 ATG |
| SA2496 | NWMN_2607 |  | hypothetical protein | 0.4 | 0.4 |  |  |
| SAS052 | NWMN_1613 | *rpsD* | 30S ribosomal protein S4 | 0.6 | 0.5 |  |  |
| SAS093 | NWMN_2614 | *rpmH* | 50S ribosomal protein L34 | 0.5 | 0.6 |  |  |

^a^ Cellular main roles are in accordance with the N315 annotation of the DOGAN website [26] and/or the KEGG website [27].

^b^ Comparison of gene expression of wild type (wt) and Δ*ccpA* mutant (mut) at OD600 1 (T0) and 30 min later (T30). Genes with a wt/mut ratio of ≤ 0.5 were considered to be regulated.

^c^ *cre*-site according to Miwa et *al.* [7] allowing up to two mismatches. Palindromic parts are underlined.
