# Supplementary material for: Effect of a glucose impulse on the CcpA regulon in Staphylococcus aureus
Source: BMC Microbiol. 2009 May 18;9:95. doi: 10.1186/1471-2180-9-95 (PMC2697999; doi:10.1186/1471-2180-9-95)
Supplement: Additional file 2 — Genes with higher expression in wild-type versus ΔccpA mutant. The table represents genes showing a higher gene expression in the wild-type than the ΔccpA mutant (wt/mutant ratio ≥ 2.0). Cells were grown in LB, without glucose addition. [file 1471-2180-9-95-S2.doc]

### Additional file 2 – Genes with higher expression in wild-type versus Δ*ccpA* mutant

| ID | |  |  | wt/mut | wt/mut |  |  |
| --- | --- | --- | --- | --- | --- | --- | --- |
| N315 | Newman | common | Product^a^ | T0^b^ | T30^b^ | *cre*^c^ | Position |
| SA0084 | NWMN_0029 |  | hypothetical protein | 4.4 | 3.1 |  |  |
| SA0098 | NWMN_0047 |  | similar to aminoacylase | 2.0 | 2.4 |  |  |
| SA0105 | NWMN_0053 |  | hypothetical protein | 2.2 | 1.8 |  |  |
| SA0144 | NWMN_0095 | *capA* | capsular polysaccharide synthesis enzyme Cap5A | 4.5 | 7.0 |  |  |
| SA0145 | NWMN_0096 | *capB* | capsular polysaccharide synthesis enzyme Cap5B | 4.6 | 6.7 |  |  |
| SA0146 | NWMN_0097 | *capC* | capsular polysaccharide synthesis enzyme Cap8C | 4.5 | 7.5 |  |  |
| SA0147 | NWMN_0098 | *capD* | capsular polysaccharide synthesis enzyme Cap5D | 3.8 | 7.3 |  |  |
| SA0148 | NWMN_0099 | *capE* | capsular polysaccharide synthesis enzyme Cap8E | 2.6 | 6.2 |  |  |
| SA0149 | NWMN_0100 | *capF* | capsular polysaccharide synthesis enzyme Cap5F | 2.9 | 7.5 |  |  |
| SA0150 | NWMN_0101 | *capG* | capsular polysaccharide synthesis enzyme Cap5G | 2.8 | 7.8 |  |  |
| SA0151 | NWMN_0102 | *capH* | capsular polysaccharide synthesis enzyme O-acetyl transferase Cap5H | 3.1 | 8.6 |  |  |
| SA0152 | NWMN_0103 | *capI* | capsular polysaccharide synthesis enzyme Cap5I | 2.0 | 5.1 |  |  |
| SA0153 | NWMN_0104 | *capJ* | capsular polysaccharide synthesis enzyme Cap5J | 2.1 | 4.6 |  |  |
| SA0154 | NWMN_0105 | *capK* | capsular polysaccharide synthesis enzyme Cap5K | 2.1 | 3.5 |  |  |
| SA0155 | NWMN_0106 | *capL* | capsular polysaccharide synthesis enzyme Cap5L | 2.0 | 3.5 |  |  |
| SA0156 | NWMN_0107 | *capM* | capsular polysaccharide synthesis enzyme Cap5M | 2.1 | 3.8 |  |  |
| SA0157 | NWMN_0108 | *capN* | capsular polysaccharide synthesis enzyme Cap5N | 2.3 | 3.9 |  |  |
| SA0158 | NWMN_0109 | *capO* | capsular polysaccharide synthesis enzyme Cap8O | 1.9 | 3.7 |  |  |
| SA0163 | NWMN_0114 | *entB* | similar to cation-efflux system membrane protein CzcD | 2.5 | 2.4 |  |  |
| SA0181 | NWMN_0131 | *ipdC* | similar to isochorismatase | 2.5 | 3.1 | ATTGTTAGCGTTTTCAGA |  |
| SA0182 | NWMN_0132 |  | similar to indole-3-pyruvate decarboxylase | 2.2 | 2.6 | ATTGTTAGCGTTTTCAGA | -81 ATG |
| SA0208 | NWMN_0153 |  | maltose/maltodextrin transport permease homologue | 2.2 | 2.2 |  |  |
| SA0209 | NWMN_0154 |  | maltose/maltodextrin transport permease homologue | 2.9 | 2.3 |  |  |
| SA0210 | NWMN_0155 | *gatC* | similar to NADH-dependent dehydrogenase | 2.8 | 2.4 |  |  |
| SA0238 | NWMN_0182 |  | probable PTS galacitol-specific enzyme IIC component | 2.5 | 2.0 |  |  |
| SA0239 | NWMN_0183 |  | sorbitol dehydrogenase | 2.9 | 2.3 |  |  |
| SA0317 | NWMN_0321 |  | similar to dihydroflavonol-4-reductase | 2.8 | 2.4 |  |  |
| SA0318 | NWMN_0322 |  | similar to transport protein SgaT | 8.1 | 2.5 |  |  |
| SA0326 | NWMN_0330 |  | conserved hypothetical protein | 2.4 | 2.4 | TATGAAAGCGTTGTCAAT | +230 ATG |
| SA0327 | NWMN_0331 |  | conserved hypothetical protein | 2.0 | 2.3 | TATGAAAGCGTTGTCAAT |  |
| SA0360 | NWMN_0366 |  | conserved hypothetical protein | 2.3 | 2.8 |  |  |
| SA0400 | NWMN_0404 | *lpl4* | staphylococcal tandem lipoprotein | 1.9 | 2.4 |  |  |
| SA0528 | NWMN_0533 |  | similar to hexulose-6-phosphate synthase | 2.3 | 2.7 |  |  |
| SA0529 | NWMN_0534 |  | conserved hypothetical protein | 2.5 | 2.9 |  |  |
| SA0531 | NWMN_0536 | *proP* | proline/betaine transporter homologue | 1.9 | 2.0 |  |  |
| SA0534 | NWMN_0539 | *vraB* | acetyl-CoA C-acetyltransferase | 2.2 | 2.3 |  |  |
| SA0549 | NWMN_0555 | *mvaK2* | phosphomevalonate kinase | 2.4 | 2.1 |  |  |
| SA0658 | NWMN_0672 |  | similar to plant-metabolite dehydrogenases | 2.1 | 2.1 |  |  |
| SA0659 | NWMN_0673 |  | similar to CsbB stress response protein | 2.6 | 3.1 |  |  |
| SA0665 | NWMN_0679 |  | coenzyme PQQ synthesis homologue | 2.0 | 1.8 |  |  |
| SA0666 | NWMN_0680 |  | 6-pyruvoyl tetrahydrobiopterin synthase homologue | 2.3 | 2.0 |  |  |
| SA0721 | NWMN_0734 |  | conserved hypothetical protein | 2.5 | 2.1 |  |  |
| SA0722 | NWMN_0735 |  | conserved hypothetical protein | 2.1 | 1.9 |  |  |
| SA0740 | NWMN_0754 |  | hypothetical protein | 2.8 | 2.5 |  |  |
| SA0741 | NWMN_0755 |  | conserved hypothetical protein | 2.0 | 2.1 |  |  |
| SA0751 | NWMN_0766 |  | hypothetical protein | 1.9 | 2.1 |  |  |
| SA0755 | NWMN_0771 |  | similar to general stress protein 170 | 3.0 | 4.1 |  |  |
| SA0760 | NWMN_0776 |  | glycine cleavage system protein H homologue | 1.8 | 2.1 | AATGTAAGCGTTTACTAA | -135 TTG |
| SA0768 | NWMN_0778 |  | conserved hypothetical protein | 4.0 | 3.3 |  |  |
| SA0780 | NWMN_0791 |  | similar to hemolysin | 3.0 | 2.8 |  |  |
| SA0781 | NWMN_0792 |  | similar to 2-nitropropane dioxygenase | 3.2 | 2.3 |  |  |
| SA0802 | NWMN_0811 |  | similar to NADH dehydrogenase | 1.9 | 2.2 |  |  |
| SA0830 | NWMN_0840 |  | conserved hypothetical protein | 2.3 | 2.7 |  |  |
| SA0848 | NWMN_0859 | *oppF* | oligopeptide transport system ATP-binding protein OppF homologue | 2.3 | 1.8 |  |  |
| SA0849 | NWMN_0860 |  | hypothetical protein, similar to peptide binding protein OppA | 2.7 | 2.0 |  |  |
| SA0905 | NWMN_0922 | *atl* | autolysin (N-acetylmuramyl-L-alanine amidase and endo-b-N-acetylglucosaminidase) | 2.2 | 2.9 |  |  |
| SA1155 | NWMN_1230 |  | cardiolipin synthetase homologue | 2.2 | 2.1 |  |  |
| SA1184 | NWMN_1263 | *citB* | aconitate hydratase | 2.3 | 2.4 |  |  |
| SA1218 | NWMN_1297 | *pstB* | phosphate ABC transporter, ATP-binding protein (pstB) | 2.0 | 3.1 |  |  |
| SA1219 | NWMN_1298 |  | similar to phosphate ABC transporter | 1.8 | 3.7 |  |  |
| SA1365 | NWMN_1439 |  | glycine dehydrogenase (decarboxylating) subunit 2 homologue | 2.1 | 2.7 |  |  |
| SA1366 | NWMN_1440 |  | glycine dehydrogenase (decarboxylating) subunit 1 | 2.0 | 2.3 |  |  |
| SA1367 | NWMN_1441 |  | aminomethyltransferase | 2.2 | 2.4 | CTTGAATGCGATTTCATT | -240 ATG |
| SA1432 | NWMN_1505 |  | conserved hypothetical protein | 6.7 | 4.1 |  |  |
| SA1433 | NWMN_1506 |  | conserved hypothetical protein | 5.1 | 2.6 |  |  |
| SA1435 | NWMN_1508 |  | similar to acetyl-CoA carboxylase (biotin carboxyl carrier subunit), accB homologue | 3.6 | 2.1 |  |  |
| SA1436 | NWMN_1509 |  | conserved hypothetical protein | 3.9 | 2.2 |  |  |
| SA1443 | NWMN_1516 |  | conserved hypothetical protein | 2.1 | 2.5 |  |  |
| SA1517 | NWMN_1587 | *citC* | isocitrate dehyrogenase | 3.0 | 4.5 | TGTGAAAGCCATTTCATA |  |
| SA1518 | NWMN_1588 | *citZ* | citrate synthase II | 3.4 | 3.5 | TGTGAAAGCCATTTCATA | -27 ATG |
| SA1528 | NWMN_1600 |  | conserved hypothetical protein | 2.0 | 3.7 |  |  |
| SA1529 | NWMN_1601 |  | conserved hypothetical protein | 1.9 | 2.0 |  |  |
| SA1557 | NWMN_1629 | *ccpA* | catabolite control protein A | 43.8 | 73.9 |  |  |
| SA1606 | NWMN_1678 |  | plant metabolite dehydrogenase homologue | 1.9 | 2.1 |  |  |
| SA1692 | NWMN_1767 |  | conserved hypothetical protein | 2.4 | 2.7 |  |  |
| SA1733 | NWMN_1855 |  | conserved hypothetical protein | 2.8 | 2.2 |  |  |
| SA1774 | NWMN_1896 |  | hypothetical protein [Bacteriophage phiN315] | 2.0 | 1.9 |  |  |
| SA1814 | NWMN_1929 |  | similar to succinyl-diaminopimelate desuccinylase | 3.2 | 3.4 |  |  |
| SA1844 | NWMN_1946 | *agrA* | accessory gene regulator A | 2.3 | 1.8 |  |  |
| SA1924 | NWMN_2026 |  | similar to aldehyde dehydrogenase | 2.9 | 2.2 |  |  |
| SA1946 | NWMN_2048 |  | conserved hypothetical protein | 2.2 | 2.3 |  |  |
| SA1981 | NWMN_2080 |  | conserved hypothetical protein | 2.6 | 2.2 |  |  |
| SA2006 | NWMN_2109 |  | similar to MHC class II analog | 3.7 | 6.2 |  |  |
| SA2082 | NWMN_2188 | *ureA* | urease gamma subunit | 2.7 | 3.5 | TTTGTTTTCGATTAGATT | -139 TTG |
| SA2083 | NWMN_2189 | *ureB* | urease beta subunit | 3.8 | 4.2 |  |  |
| SA2084 | NWMN_2190 | *ureC* | urease alpha subunit | 2.3 | 2.6 |  |  |
| SA2086 | NWMN_2192 | *ureF* | urease accessory protein UreF | 2.0 | 2.1 |  |  |
| SA2119 | NWMN_2229 |  | similar to dehydrogenase | 2.3 | 2.4 |  |  |
| SA2125 | NWMN_2235 |  | similar to formiminoglutamase | 2.6 | 2.8 |  |  |
| SA2170 | NWMN_2282 |  | similar to general stress protein 26 | 1.8 | 2.1 |  |  |
| SA2204 | NWMN_2315 |  | phosphoglycerate mutase, pgm homologue | 2.5 | 1.8 | TATGAAGACGTTTAAATA | -306 ATG |
| SA2220 | NWMN_2331 |  | conserved hypothetical protein | 2.1 | 1.9 |  |  |
| SA2243 | NWMN_2353 |  | similar to ABC transporter (ATP-binding protein) | 2.0 | 2.1 |  |  |
| SA2260 | NWMN_2369 |  | similar to glucose 1-dehydrogenase | 2.9 | 2.5 |  |  |
| SA2262 | NWMN_2371 |  | conserved hypothetical protein | 2.8 | 2.8 |  |  |
| SA2267 | NWMN_2376 |  | hypothetical protein | 2.3 | 2.2 |  |  |
| SA2309 | NWMN_2419 |  | conserved hypothetical protein | 1.9 | 2.2 |  |  |
| SA2331 | NWMN_2442 |  | hypothetical protein | 1.9 | 2.3 |  |  |
| SA2350 | NWMN_2463 |  | conserved hypothetical protein | 2.6 | 1.8 |  |  |
| SA2366 | NWMN_2479 |  | conserved hypothetical protein | 3.0 | 3.1 |  |  |
| SA2367 | NWMN_2480 |  | conserevd hypothetical protein | 2.6 | 3.0 |  |  |
| SA2374 | NWMN_2487 |  | conserved hypothetical protein | 1.9 | 2.1 |  |  |
| SA2479 | NWMN_2585 |  | conserved hypothetical protein | 2.0 | 3.0 |  |  |
| SA2485 | NWMN_2591 |  | hypothetical protein | 3.4 | 3.6 |  |  |
| SA2488 | NWMN_2594 |  | hypothetical protein | 2.6 | 1.8 |  |  |
| SAS022 | NWMN_0777 |  | truncated conserved hypothetical protein | 2.0 | 2.4 |  |  |
| SAS023 | NWMN_0779 |  | similar to thioredoxin | 3.5 | 2.8 |  |  |
| SAS056 | NWMN_1861 |  | hypothetical protein | 2.1 | 2.5 |  |  |

^a^ Cellular main roles are in accordance with the N315 annotation of the DOGAN website [26] and/or the KEGG website [27].

^b^ Comparison of gene expression of wild type (wt) and Δ*ccpA* mutant (mut) at OD600 1 (T0) and 30 min later (T30). Genes with a wt/mut ratio of ≥2 were considered to be regulated.

^c^ *cre*-site according to Miwa et *al.* [7] allowing up to two mismatches. Palindromic parts are underlined.
