# Supplementary material for: Effect of a glucose impulse on the CcpA regulon in Staphylococcus aureus
Source: BMC Microbiol. 2009 May 18;9:95. doi: 10.1186/1471-2180-9-95 (PMC2697999; doi:10.1186/1471-2180-9-95)
Supplement: Additional file 3 — CcpA-dependent down-regulation by glucose. The table shows genes found to be subject to down-regulation by glucose in a CcpA-dependent manner (with/without glucose ratio of 0.5 or lower in wild-type, with/without glucose ratio of approximately 1, but below 2 in the mutant). [file 1471-2180-9-95-S3.doc]

### Additional file 3 – CcpA-dependent down-regulation by glucose

| ID | |  |  | wt | mut |  |  |
| --- | --- | --- | --- | --- | --- | --- | --- |
| N315 | Newman | common | Product^a^ | **+/-^b^** | **+/-^b^** | *cre*^c^ | Position |
| SA0008 | NWMN_0007 | *hutH* | histidine ammonia-lyase | 0.5 | 1.1 |  |  |
|  |  |  |  |  |  |  |  |
| SA0016 | NWMN_0016 | *purA* | adenylosuccinate synthase | 0.3 | 1.0 |  |  |
|  |  |  |  |  |  |  |  |
| SA0100 | NWMN_0049 |  | similar to Na+ Pi-cotransporter | 0.2 | 1.7 |  |  |
|  |  |  |  |  |  |  |  |
| SA0107 | NWMN_0055 | *spa* | immunoglobulin G binding protein A precursor | 0.2 | 1.1 | TATTAAACCGCTTTCATT | -221 TTG |
|  |  |  |  |  |  |  |  |
| SA0162 | NWMN_0113 | *aldA* | aldehyde dehydrogenase homologue | 0.3 | 1.0 | AATGTAAACGCTTACTAT | - 87 ATG |
|  |  |  |  |  |  |  |  |
| SA0181 | NWMN_0131 | *entB* | similar to isochorismatase | 0.2 | 1.2 |  |  |
| SA0182 | NWMN_0132 | *ipdC* | similar to indole-3-pyruvate decarboxylase | 0.1 | 1.0 | ATTGTTAGCGTTTTCAGA | -81 ATG |
|  |  |  |  |  |  |  |  |
| SA0184 | NWMN_0134 |  | conserved hypothetical protein | 0.3 | 1.2 | AATGTAAGCGATTACACA | -46 TTG |
| SA0185 | NWMN_0135 |  | similar to glucokinase regulator protein | 0.2 | 1.2 |  |  |
| SA0186 | NWMN_0136 |  | similar to sucrose phosphotransferase enzyme II | 0.4 | 1.2 |  |  |
| SA0187 | NWMN_0137 |  | RpiR family transcriptional regulator | 0.2 | 1.2 |  |  |
|  |  |  |  |  |  |  |  |
| SA0224 | NWMN_0168 |  | 3-hydroxyacyl-CoA dehydrogenase FadB homologue | 0.4 | 1.1 |  |  |
|  |  |  |  |  |  |  |  |
| SA0299 | NWMN_0253 |  | similar to carbohydrate kinase, PfkB family | 0.6^§^ | 1.7 | AATGTAAGCGTTTACAAC | -76 ATG |
| SA0300 | NWMN_0253 |  | truncated hypothetical protein | 0.6^§^ | 1.5 |  |  |
| SA0301 | NWMN_0254 |  | conserved hypothetical protein | 0.3 | 1.7 |  |  |
| SA0302 | NWMN_0255 |  | probable pyrimidine nucleoside transport protein | 0.4 | 1.8 |  |  |
|  |  |  |  |  |  |  |  |
| SA0395 | NWMN_0402 |  | similar to functionally unknown protein | 0.4 | 0.9 |  |  |
|  |  |  |  |  |  |  |  |
| SA0477 | NWMN_0481 |  | conserved hypothetical protein | 0.3 | 1.3 |  |  |
| SA0478 | NWMN_0482 |  | conserved hypothetical protein | 0.3 | 1.2 |  |  |
|  |  |  |  |  |  |  |  |
| SA0605 | NWMN_619 |  | similar to dihydroxyacetone kinase | 0.6^§^ | 1.0 | TATGATAGCGCATTCATT | -44 ATG |
| SA0606 | NWMN_620 |  | conserved hypothetical protein | 0.5 | 1.0 |  |  |
| SA0607 | NWMN_0621 |  | conserved hypothetical protein | 0.5 | 1.0 |  |  |
|  |  |  |  |  |  |  |  |
| SA0620 | NWMN_0634 |  | secretory antigen SsaA homologue | 0.4 | 0.9 |  |  |
|  |  |  |  |  |  |  |  |
| SA0748 | NWMN_0762 |  | hypothetical protein | 0.5 | 1.2 |  |  |
| SA0749 | NWMN_0763 |  | hypothetical protein | 0.4 | 1.3 |  |  |
|  |  |  |  |  |  |  |  |
| SA0760 | NWMN_0776 |  | glycine cleavage system protein H homologue | 0.3 | 1.0 | AATGTAAGCGTTTACTAA | -135 TTG |
|  |  |  |  |  |  |  |  |
| SA0818 | NWMN_0827 | *rocD* | ornithine aminotransferase | 0.3 | 1.0 | AATGTAAGGGTTTTCAAA | - 133 ATG |
| SA0819 | NWMN_0828 | *gudB* | NAD-specific glutamate dehydrogenase | 0.2 | 1.1 | TTTGTAAGGGCTTTAAAA | - 24 TTG |
|  |  |  |  |  |  | TTTGAAAGCGAAATCATT | overlaps TTG |
|  |  |  |  |  |  |  |  |
| SA0830 | NWMN_0840 |  | conserved hypothetical protein | 0.5 | 1.2 |  |  |
|  |  |  |  |  |  |  |  |
| SA0841 | NWMN_0851 |  | truncated MHC class II analog protein | 0.4 | 0.9 | AATTATAGCTTTTACATT | -120 ATG |
|  |  |  |  |  |  |  |  |
| SA0905 | NWMN_0922 | *atl* | autolysin (N-acetylmuramyl-L-alanine amidase and endo-b-N-acetylglucosaminidase) | 0.4 | 1.1 |  |  |
|  |  |  |  |  |  |  |  |
| SA0915 | NWMN_0932 | *folD* | FolD bifunctional protein | 0.4 | 1.3 |  |  |
|  |  |  |  |  |  |  |  |
| SA1019 | NWMN_1086 |  | acetyltransferase, GNAT family protein | 0.3 | 1.1 |  |  |
|  |  |  |  |  |  |  |  |
| SA1041 | NWMN_1109 | *pyrR* | pyrimidine operon repressor chainA | 0.4 | 1.1 |  |  |
|  |  |  |  |  |  |  |  |
| SA1088 | NWMN_1155 | *sucC* | succinyl-CoA synthetase (beta subunit) | 0.2 | 1.4 |  |  |
| SA1089 | NWMN_1156 | *sucD* | succinyl-CoA synthetase (alpha subunit) | 0.2 | 1.5 | TACAATAGCGCTTACATT | - 48 ATG |
|  |  |  |  |  |  |  |  |
| SA1140 | NWNM_1207 | *glpF* | glycerol uptake facilitator | 1.3^§^ | 0.8 | ATTGACAACGCTTTCATA | -116 ATG |
| SA1141 | NWMN_1208 | *glpK* | glycerol kinase | 0.4 | 0.9 |  |  |
|  |  |  |  |  |  |  |  |
| SA1149 | NWMN_1216 | *glnR* | glutamine synthetase repressor | 0.4 | 1.2 |  |  |
| SA1150 | NWMN_1217 | *glnA* | glutamine synthetase | 0.5 | 1.3 |  |  |
|  |  |  |  |  |  |  |  |
| SA1172 | NWMN_1249 |  | guanosine monophosphate reductase | 0.5 | 0.8 |  |  |
|  |  |  |  |  |  |  |  |
| SA1184 | NWMN_1263 | *citB* | aconitate hydratase | 0.1 | 1.1 |  |  |
|  |  |  |  |  |  |  |  |
| SA1244 | NWMN_1325 | *odhB* | dihydrolipoamide succinyltransferase | 0.1 | 1.1 |  |  |
| SA1245 | NWNM_1326 | *odhA* | 2-oxoglutarate dehydrogenase E1 | 0.5 | 1.2 | ATTGTAAGCGTTTCAACA | - 19 ATG |
|  |  |  |  |  |  |  |  |
| SA1365 | NWMN_1439 |  | glycine dehydrogenase (decarboxylating) subunit 2 homologue | 0.1 | 1.1 |  |  |
| SA1366 | NWMN_1440 |  | glycine dehydrogenase (decarboxylating) subunit 1 | 0.1 | 1.2 |  |  |
| SA1367 | NWMN_1441 |  | aminomethyltransferase | 0.2 | 1.4 |  |  |
|  |  |  |  |  |  |  |  |
| SA1432 | NWMN_1505 |  | conserved hypothetical protein | 0.1 | 1.0 |  |  |
| SA1433 | NWMN_1506 |  | LamB/YcsF family protein | 0.1 | 1.1 |  |  |
| SA1434 | NWMN_1507 | *accC* | acetyl-CoA biotin carboxylase | 0.1 | 1.0 |  |  |
| SA1435 | NWMN_1508 | *accB* | acetyl-CoA carboxylase, biotin carboxyl carrier | 0.2 | 0.8 |  |  |
| SA1436 | NWMN_1509 |  | urea amidolyase-related protein | 0.2 | 0.8 |  |  |
| SA1437 | NWMN_1510 |  | similar to allophanate hydrolase subunit 1 | 0.3 | 0.6 | AATGAAAAGGTATTCAAT | -34 GTG |
|  |  |  |  |  |  | ATTGTATGCGATTTGAAT | -86 ATG |
|  |  |  |  |  |  |  |  |
| SA1516 | NWMN_1586 | *phoP* | alkaline phosphatase synthesis transcriptional regulatory protein | 0.4 | 1.6 |  |  |
|  |  |  |  |  |  |  |  |
| SA1517 | NWMN_1587 | *citC* | isocitrate dehyrogenase | 0.1 | 1.0 |  |  |
| SA1518 | NWMN_1588 | *citZ* | citrate synthase II | 0.1 | 0.9 | TGTGAAAGCCATTTCATA | -27 ATG |
|  |  |  |  |  |  |  |  |
| SA1528 | NWMN_1600 |  | universal stress protein family protein | 0.3 | 1.0 |  |  |
|  |  |  |  |  |  |  |  |
| SA1531 | NWMN_1603 | *ald* | alanine dehydrogenase | 0.1 | 1.0 | TTTGATTGCGCTTTCAAA | -36 ATG |
|  |  |  |  |  |  |  |  |
| SA1553 | NWMN_1625 | *fhs* | formyltetrahydrofolate synthetase | 0.4 | 1.1 |  |  |
|  |  |  |  |  |  |  |  |
| SA1609 | NWMN_1681 | *pckA* | phosphoenolpyruvate carboxykinase [ATP] | 0.2 | 0.7 | AATGTAAAGGCTTACATT | -27 ATG |
|  |  |  |  |  |  |  |  |
| SA1617 | NWMN_1688 |  | similar to functionally unknown protein | 0.3 | 1.3 | TTTAAAAACTTTTTCAAA | -274 ATG |
| SA1618 | NWMN_1689 |  | conserved hypothetical protein | 0.4 | 1.3 |  |  |
|  |  |  |  |  |  |  |  |
| SA1848 | NWMN_1950 | *nrgA* | probable ammonium transporter | 0.4 | 0.8 |  |  |
|  |  |  |  |  |  |  |  |
| SA1889 | NWMN_1990 |  | hypothetical protein | 0.4 | 1.0 |  |  |
| SA1890 | NWMN_1991 |  | conserved hypothetical protein | 0.5 | 1.0 |  |  |
|  |  |  |  |  |  |  |  |
| SA1900 | NWMN_2002 |  | conserved hypothetical protein | 0.4 | 1.0 |  |  |
|  |  |  |  |  |  |  |  |
| SA1932 | NWMN_2034 |  | similar to hypothetical protein T13D8.31 - Arabidopsis thaliana | 0.5 | 0.8 |  |  |
| SA1938 | NWMN_2040 | *pdp* | pyrimidine nucleoside phosphorylase | 0.5 | 1.0 |  |  |
|  |  |  |  |  |  |  |  |
| SA1968 | NWMN_2065 | *arg* | arginase | 0.4 | 1.0 | ATGGTAAGCGCATACATT | -35 ATG |
|  |  |  |  |  |  |  |  |
| SA2121 | NWMN_2231 | *hutI* | imidazolonepropionase | 0.5 | 1.1 |  |  |
| SA2122 | NWMN_2232 | *hutU* | urocanate hydratase | 0.7^§^ | 1.3 | TATGTAACCGCATACATA | -41 ATG |
|  |  |  |  |  |  |  |  |
| SA2226 | NWMN_2337 |  | amino acid permease | 0.2 | 0.9 |  |  |
| SA2227 | NWMN_2337 |  | truncated hypothetical protein, similar to D-serine/D-alanine/glycine transporter | 0.1 | 0.9 |  |  |
|  |  |  |  |  |  |  |  |
| SA2311 | NWMN_2421 |  | NAD(P)H-flavin oxidoreductase | 0.5 | 1.0 | ATTGCAAACGGATTACTT | -67 ATG |
|  |  |  |  |  |  |  |  |
| SA2341 | NWMN_2454 | *rocA* | 1-pyrroline-5-carboxylate dehydrogenase | 0.2 | 1.2 | AATGAAAGCGATTGCAAA | -96 ATG |
|  |  |  |  |  |  |  |  |
| SA2353 | NWMN_2466 |  | similar to secretory antigen precursor SsaA | 0.5 | 1.0 |  |  |
|  |  |  |  |  |  |  |  |
| SA2356 | NWMN_2469 | *isaA* | immunodominant antigen A | 0.4 | 0.8 |  |  |
|  |  |  |  |  |  |  |  |
| SA2366 | NWMN_2479 |  | conserved hypothetical protein | 0.4 | 1.1 |  |  |
| SA2367 | NWMN_2480 |  | conserevd hypothetical protein | 0.4 | 1.0 |  |  |
|  |  |  |  |  |  |  |  |
| SA2378 | NWMN_2491 |  | conserved hypothetical protein | 0.5 | 1.2 |  |  |
|  |  |  |  |  |  |  |  |
| SA2420 | NWMN_2526 | *phoB* | alkaline phosphatase (EC 3.1.3.1) III precursor | 0.2 | 1.4 |  |  |
|  |  |  |  |  |  |  |  |
| SA2479 | NWMN_2585 |  | conserved hypothetical protein | 0.5 | 1.3 |  |  |
|  |  |  |  |  |  |  |  |
| SA2485 | NWMN_2591 |  | hypothetical protein | 0.4 | 1.4 |  |  |
|  |  |  |  |  |  |  |  |
| SAS022 | NWMN_0777 |  | truncated conserved hypothetical protein | 0.4 | 1.1 |  |  |
|  |  |  |  |  |  |  |  |
| SAS068 | NWMN_1989 |  | hypothetical protein | 0.5 | 0.9 |  |  |
|  |  |  |  |  |  |  |  |
| SAS074 | NWMN_2074 |  | conserved hypothetical protein | 0.5 | 0.6 | CTTGAAAACGATTACAAA | -47 ATG |

^a^ Cellular main roles are in accordance with the N315 annotation of the DOGAN website [26] and/or the KEGG website [27].

^b^ Comparison of gene expression with (+) and without (-) glucose. Genes with a +/- glucose ratio of ≤ 0.5 in the wild-type were considered to be regulated.

^c^ *cre*-site according to Miwa et *al.* [7] allowing up to two mismatches. Palindromic parts are underlined.

^§^ Genes with regulation above threshold, which were included in the list because they were part of a putative operon.
