# Supplementary material for: Effect of a glucose impulse on the CcpA regulon in Staphylococcus aureus
Source: BMC Microbiol. 2009 May 18;9:95. doi: 10.1186/1471-2180-9-95 (PMC2697999; doi:10.1186/1471-2180-9-95)
Supplement: Additional file 4 — CcpA-dependent up-regulation by glucose. The table shows genes found to be subject to up-regulation by glucose in a CcpA-dependent manner (with/without glucose ratio of 2 or higher in wild-type, with/without glucose ratio of approximately 1, but below 2 in the mutant). [file 1471-2180-9-95-S4.doc]

### Additional file 4 – CcpA-dependent up-regulation by glucose

| ID | |  |  | wt | mut |  |  |
| --- | --- | --- | --- | --- | --- | --- | --- |
| N315 | Newman | common | Product^a^ | **+/-^b^** | **+/-^b^** | *cre*^c^ | Position |
| SA0009 | NWMN_0008 | *serS* | seryl-tRNA synthetase | 2.6 | 0.8 |  |  |
|  |  |  |  |  |  |  |  |
| SA0143 | NWMN_0094 | *adhE* | alcohol-acetaldehyde dehydrogenase | 2.3 | 1.2 |  |  |
|  |  |  |  |  |  |  |  |
| SA0166 | NWMN_0116 |  | similar to nitrate transporter | 2.8 | 1.1 |  |  |
| SA0167 | NWMN_0117 |  | similar to membrane lipoprotein SrpL | 2.8 | 1.6 | TATGGAAACATTTGCATT | -50 ATG |
| SA0168 | NWMN_0118 |  | similar to probable permease of ABC transporter | 2.3 | 1.1 |  |  |
|  |  |  |  |  |  |  |  |
| SA0214 | NWMN_0158 | *uhpT* | hexose phosphate transport protein | 2.1 | 1.1 |  |  |
|  |  |  |  |  |  |  |  |
| SA0218 | NWMN_0162 | *pflB* | formate acetyltransferase | 2.7 | 1.6 | TATGAAAACGTTAACATA | -42 ATG |
| SA0219 | NWMN_0163 | *pflA* | formate acetyltransferase activating enzyme | 2.3 | 1.6 |  |  |
|  |  |  |  |  |  |  |  |
| SA0230 | NWMN_0174 |  | conserved hypothetical protein | 2.3 | 1.9 |  |  |
|  |  |  |  |  |  |  |  |
| SA0335 | NWMN_0340 |  | twin-arginine translocation protein TatA | 2.2 | 1.4 |  |  |
|  |  |  |  |  |  |  |  |
| SA0373 | NWMN_0378 | *xprT* | xanthine phosphoribosyltransferase | 5.3 | 1.1 |  |  |
| SA0374 | NWMN_0379 | *pbuX* | xanthine permease | 7.2 | 1.1 |  |  |
| SA0375 | NWMN_0380 | *guaB* | inositol-monophosphate dehydrogenase | 3.6 | 1.2 |  |  |
| SA0376 | NWMN_0381 | *guaA* | GMP synthase (glutamine-hydrolyzing) | 3.3 | 1.2 |  |  |
|  |  |  |  |  |  |  |  |
| SA0411 | NWMN_0418 | *ndhF* | NADH dehydrogenase subunit 5 | 2.5 | 0.9 |  |  |
| SA0412 | NWMN_0419 |  | conserved hypothetical protein | 2.6 | 1.1 |  |  |
|  |  |  |  |  |  |  |  |
| SA0452 | NWMN_0457 | *veg* | VEG protein homologue | 3.1 | 1.0 |  |  |
|  |  |  |  |  |  |  |  |
| SA0461 | NWMN_0466 | *mfd* | transcription-repair coupling factor | 2.1 | 1.1 |  |  |
| SA0462 | NWMN_0467 |  | similar to low temperature requirement B protein | 2.6 | 1.2 |  |  |
|  |  |  |  |  |  |  |  |
| SA0492 | NWMN_0496 |  | hypothetical protein | 2.4 | 1.0 |  |  |
|  |  |  |  |  |  |  |  |
| SA0496 | NWMN_0500 | *rplA* | 50S ribosomal protein L1 (BL1) | 2.1 | 0.9 |  |  |
|  |  |  |  |  |  |  |  |
| SA0497 | NWMN_0501 | *rplJ* | 50S ribosomal protein L10 (BL5) | 2.6 | 1.6 |  |  |
|  |  |  |  |  |  |  |  |
| SA0577 | NWMN_0592 |  | similar to FimE recombinase | 2.0 | 1.3 |  |  |
|  |  |  |  |  |  |  |  |
| SA0655 | NWMN_0669 | *fruA* | fructose specific permease | 2.4 | 1.3 | ATTGAAAAAGCATTCCAA | -116 ATG |
|  |  |  |  |  |  |  |  |
| SA0685 | NWMN_0699 | *nrdI* | NrdI protein involved in ribonucleotide reductase function | 2.1 | 1.0 |  |  |
| SA0686 | NWMN_0700 | *nrdE* | ribonuceloside diphosphate reductase major subunit | 3.2 | 1.4 |  |  |
| SA0687 | NWMN_0701 | *nrdF* | ribonucleoside-diphosphate reductase minor subunit | 1.9^§^ | 1.1 |  |  |
|  |  |  |  |  |  |  |  |
| SA0726 | NWMN_0740 | *gapR* | glycolytic operon regulator | 1.5^§^ | 1.3 |  |  |
| SA0727 | NWMN_0741 | *gap* | glyceraldehyde-3-phosphate dehydrogenase | 1.7^§^ | 1.6 |  |  |
| SA0728 | NWMN_0742 | *pgk* | phosphoglycerate kinase | 2.2 | 1.6 |  |  |
| SA0729 | NWMN_0743 | *tpi* | triosephosphate isomerase | 2.0 | 1.3 |  |  |
| SA0730 | NWMN_0744 | *pgm* | 2, 3-diphosphoglycerate-independent phosphoglycerate mutase | 1.7^§^ | 1.2 |  |  |
| SA0731 | NWMN_0745 | *eno* | enolase (2-phosphoglycerate dehydrogenase) | 1.4^§^ | 1.3 |  |  |
|  |  |  |  |  |  |  |  |
| SA0769 | NWMN_0780 |  | D-methionine transport system ATP-binding protein | 2.4 | 0.8 |  |  |
| SA0770 | NWMN_0780 |  | D-methionine transport system permease | 2.4 | 1.0 |  |  |
|  |  |  |  |  |  |  |  |
| SA0922 | NWMN_0938 | *purF* | phosphoribosylpyrophosphate amidotransferase PurF | 2.7 | 1.2 |  |  |
| SA0923 | NWMN_0939 | *purM* | phosphoribosylformylglycinamidine cyclo-ligase PurM | 3.2 | 1.1 |  |  |
| SA0924 | NWMN_0940 | *purN* | phosphoribosylglycinamide formyltransferase | 3.2 | 0.9 |  |  |
| SA0925 | NWMN_0941 | *purH* | bifunctional purine biosynthesis protein PurH | 3.9 | 0.9 |  |  |
| SA0926 | NWMN_0942 | *purD* | phosphoribosylamine-glycine ligase PurD | 3.2 | 0.9 |  |  |
|  |  |  |  |  |  |  |  |
| SA0938 | NWMN_0953 | *cydB* | cytochrome D ubiquinol oxidase subunit II homologue | 2.0 | 1.1 |  |  |
|  |  |  |  |  |  |  |  |
| SA1010 | NWMN_1076 |  | similar to exotoxin 4 | 2.3 | 0.6 |  |  |
|  |  |  |  |  |  |  |  |
| SA1200 | NWMN_1280 | *trpG* | anthranilate synthase component II | 2.3 | 1.0 |  |  |
| SA1201 | NWMN_1281 | *trpD* | anthranilate phosphoribosyltransferase | 2.0 | 1.0 |  |  |
| SA1202 | NWMN_1282 | *trpC* | indole-3-glycerol phosphate synthase | 2.0 | 0.9 |  |  |
| SA1203 | NWMN_1283 | *trpF* | phosphoribosylanthranilate isomerase | 2.2 | 1.0 |  |  |
| SA1204 | NWMN_1284 | *trpB* | tryptophan synthase beta chain | 1.8^§^ | 1.0 |  |  |
| SA1205 | NWMN_1285 | *trpA* | tryptophan synthase alpha chain | 1.4^§^ | 1.0 |  |  |
|  |  |  |  |  |  |  |  |
| SA1240 | NWMN_1321 |  | conserved hypothetical protein | 2.4 | 1.2 |  |  |
|  |  |  |  |  |  |  |  |
| SA1241 | NWMN_1322 |  | similar to nitric-oxide reductase | 2.3 | 1.2 |  |  |
|  |  |  |  |  |  |  |  |
| SA1270 | NWMN_1347 |  | similar to amino acid permease | 2.0 | 1.1 |  |  |
|  |  |  |  |  |  |  |  |
| SA1295 | NWMN_1373 |  | conserved hypothetical protein | 2.0 | 1.2 |  |  |
|  |  |  |  |  |  |  |  |
|  |  |  |  |  |  |  |  |
| SA1339 | NWMN_1415 | *malR* | maltose operon transcriptional repressor | 2.2 | 1.0 | GTTGGAAGCGTTTTCCAA  TATGCAATCGTTTGCACA | - 27 ATG  -94 ATG |
|  |  |  |  |  |  |  |  |
| SA1442 | NWMN_1515 |  | similar to caffeoyl-CoA O-methyltransferase | 2.0 | 0.8 |  |  |
|  |  |  |  |  |  |  |  |
| SA1493 | NWMN_1563 | *hemD* | uroporphyrinogen III synthase | 2.5 | 1.1 |  |  |
| SA1494 | NWMN_1564 | *hemC* | porphobilinogen deaminase | 2.8 | 0.9 |  |  |
| SA1495 | NWMN_1565 | *hemX* | HemA concentration negative effector *hemX* | 2.7 | 0.9 |  |  |
|  |  |  |  |  |  |  |  |
| SA1502 | NWMN_1572 | *rplT* | 50S ribosomal protein L20 | 1.9^§^ | 0.8 |  |  |
| SA1503 | NWMN_1573 | *rpmI* | 50S ribosomal protein L35 | 2.1 | 0.8 |  |  |
| SA1504 | NWMN_1574 | *infC* | translation initiation factor IF-3 infC | 2.4 | 0.9 |  |  |
|  |  |  |  |  |  |  |  |
| SA1506 | NWMN_1576 | *thrS* | threonyl-tRNA synthetase 1 | 2.2 | 1.4 |  |  |
|  |  |  |  |  |  |  |  |
| SA1608 | NWMN_1680 | *metK* | S-adenosylmethionine synthetase | 2.2 | 0.9 | AATGTAAGCCTTTACATT | -225 ATG |
|  |  |  |  |  |  |  |  |
| SA1700 | NWMN_1822 | *vraR* | two-component response regulator | 2.2 | 0.8 |  |  |
| SA1701 | NWMN_1823 | *vraS* | two-component sensor histidine kinase | 2.5 | 0.7 |  |  |
| SA1702 | NWMN_1824 |  | conserved hypothetical protein | 2.1 | 0.8 |  |  |
|  |  |  |  |  |  |  |  |
|  |  |  |  |  |  |  |  |
| SA1710 | NWMN_1832 |  | similar to DNA polymerase III, alpha chain PolC type | 2.1 | 0.8 |  |  |
|  |  |  |  |  |  |  |  |
| SA1849 | NWMN_1951 |  | conserved hypothetical protein | 2.6 | 1.2 |  |  |
| SA1850 | NWMN_1952 |  | conserved hypothetical protein | 2.1 | 1.3 |  |  |
|  |  |  |  |  |  |  |  |
| SA1870 | NWMN_1971 | *rsbW* | anti-sigmaB factor | 2.2 | 1.1 |  |  |
|  |  |  |  |  |  |  |  |
| SA1960 | NWMN_2057 | *mtlF* | PTS system, mannitol specific IIBC component | 6.4 | 0.2 |  |  |
|  |  |  |  |  |  |  |  |
| SA2053 | NWMN_2158 |  | glucose uptake protein homologue | 2.5 | 1.2 |  |  |
| SA2183 | NWMN_2295 | *narJ* | similar to nitrate reductase delta chain | 2.1 | 0.7 |  |  |
| SA2184 | NWMN_2296 | *narH* | nitrate reductase beta chain narH | 2.4 | 0.7 |  |  |
| SA2186 | NWMN_2298 | *nasF* | uroporphyrin-III C-methyl transferase | 3.2 | 0.9 |  |  |
| SA2187 | NWMN_2299 | *nasE* | assimilatory nitrite reductase | 3.5 | 1.0 |  |  |
| SA2188 | NWMN_2300 | *nasD* | nitrite reductase | 2.4 | 0.9 |  |  |
|  |  |  |  |  |  |  |  |
|  |  |  |  |  |  |  |  |
| SA2234 | NWMN_2344 | *opuCD* | probable glycine betaine/carnitine/choline ABC transporter (membrane part) OpuCD | 1.6^§^ | 1.2 |  |  |
| SA2235 | NWMN_2345 | *opuCC* | glycine betaine/carnitine/choline ABC transporter (osmoprotection) OpuCC | 1.9^§^ | 1.2 |  |  |
| SA2236 | NWMN_2346 | *opuCB* | probable glycine betaine/carnitine/choline ABC transporter (membrane part) OpuCB | 1.9^§^ | 1.1 |  |  |
| SA2237 | NWMN_2347 | *opuCA* | glycine betaine/carnitine/choline ABC transporter (ATP-binding) OpuCA | 2.6 | 1.0 | AATAAATTCGCTTTTAAA | -311 ATG |
|  |  |  |  |  |  |  |  |
| SA2239 | NWMN_2349 |  | similar to amino acid transporter | 2.2 | 1.1 |  |  |
|  |  |  |  |  |  |  |  |
| SA2290 | NWMN_2397 | *fnbB* | fibronectin-binding protein homologue | 2.6 | 1.5 |  |  |
|  |  |  |  |  |  |  |  |
| SA2329 | NWMN_2440 | *cidA* | conserved hypothetical protein | 3.5 | 1.4 | TATGGAAACGCTCTCTAA | -107 ATG |
|  |  |  |  |  |  |  |  |
| SA2443 | NWMN_2549 |  | similar to accessory secretory protein Asp3 | 2.0 | 1.2 |  |  |
| SA2444 | NWMN_2550 |  | similar to accessory secretory protein Asp2 | 2.3 | 1.3 |  |  |

^a^ Cellular main roles are in accordance with the N315 annotation of the DOGAN website [26] and/or the KEGG website [27].

^b^ Comparison of gene expression with (+) and without (-) glucose. Genes with a +/- glucose ratio of ≥2 in the wild-type were considered to be regulated.

^c^ *cre*-site according to Miwa et *al.* [7] allowing up to two mismatches. Palindromic parts are underlined.

^§^ Genes with regulation below threshold, which were included in the list because they were part of a putative operon.
