# Supplementary material for: Effect of a glucose impulse on the CcpA regulon in Staphylococcus aureus
Source: BMC Microbiol. 2009 May 18;9:95. doi: 10.1186/1471-2180-9-95 (PMC2697999; doi:10.1186/1471-2180-9-95)
Supplement: Additional file 5 — Primers used for the construction of DIG-labelled DNA probes. [file 1471-2180-9-95-S5.doc]

### Additional file 5 – Primers used for the construction of DIG-labelled DNA probes

| Primer | Sequence (5’-3’) | Source or reference |
| --- | --- | --- |
| *aldA+* | ACAATCGAAGTGACTAATCC | This study |
| *aldA-* | CGTACGATACTCATTGTGTC | This study |
| *arg+* | TGATGCAGTAATTCCAATGC | This study |
| *arg-* | AATATACCTGAAGAGTCACC | This study |
| *argF+* | CTTATTAACACTCTCCGAGG | This study |
| *argF-* | TTCTGTTCTCAGCTTCTTGG | This study |
| *pflAB+* | GTAACTGAATCTATCGGTGG | This study |
| *pflAB-* | CAATCGTAAGGGATCTTAGC | This study |
| *rocA+* | GTATTGTCTACAGTATTGCC | This study |
| *rocA-* | TTAGAAGAAGCAGGATTACC | This study |
| *rocD+* | TAACTTAGGTGAATGGTACG | This study |
| *rocD-* | TCTAATGCAGCAATTGATGC | This study |
| SA0301+ | AATAATGGTGCCATTCCAGC | This study |
| SA0301- | GATAAGGCATGCTCATATGG | This study |
| SA0318+ | TTAGGATACCATTTAGAGCG | This study |
| SA0318- | ATTCAAGGTGTGACATTTGC | This study |
| SA0768+ | ACTGTCAAGTTGCGAATTGC | This study |
| SA0768- | AACCTTACAAGTACCACACG | This study |
